# Potential of Soft-Shelled Rugby Headgear to Lower Regional Brain Strain Metrics During Standard Drop Tests

**DOI:** 10.1186/s40798-024-00744-2

**Published:** 2024-09-27

**Authors:** Danyon Stitt, Natalia Kabaliuk, Keith Alexander, Nick Draper

**Affiliations:** 1 Department of Mechanical Engineering, University of Canterbury, Christchurch, 8041 New Zealand; 2 University of Canterbury, Sports Health and Rehabilitation Research Center (SHARRC), Christchurch, 8041 New Zealand; 3 Faculty of Health, University of Canterbury, Christchurch, 8041 New Zealand

**Keywords:** Rugby, Headgear, Brain strain, Concussion, Rotational

## Abstract

**Background:**

The growing concern for player safety in rugby has led to an increased focus on head impacts. Previous laboratory studies have shown that rugby headgear significantly reduces peak linear and rotational accelerations compared to no headgear. However, these metrics may have limited relevance in assessing the effectiveness of headgear in preventing strain-based brain injuries like concussions. This study used an instantaneous deep-learning brain injury model to quantify regional brain strain mitigation of rugby headgear during drop tests. Tests were conducted on flat and angled impact surfaces across different heights, using a Hybrid III headform and neck.

**Results:**

Headgear presence generally reduced the peak rotational velocities, with some headgear outperforming others. However, the effect on peak regional brain strains was less consistent. Of the 5 headgear tested, only the newer models that use open cell foams at densities above 45 kg/m^3^ consistently reduced the peak strain in the cerebrum, corpus callosum, and brainstem. The 3 conventional headgear that use closed cell foams at or below 45 kg/m^3^ showed no consistent reduction in the peak strain in the cerebrum, corpus callosum, and brainstem.

**Conclusions:**

The presence of rugby headgear may be able to reduce the severity of head impact exposure during rugby. However, to understand how these findings relate to brain strain mitigation in the field, further investigation into the relationship between the impact conditions in this study and those encountered during actual gameplay is necessary.

**Supplementary Information:**

The online version contains supplementary material available at 10.1186/s40798-024-00744-2.

## Background

Within contact and collision sports, head impact exposure has been linked to adverse mental and physical health outcomes, even in the absence of a formally diagnosed traumatic brain injury (TBI) [1–3]. Concussion, a complex form of TBI [4], is especially prevalent in rugby [5–7], where players experience an average of 14–52 significant head impacts per game [8–10]. In terms of protective equipment, World Rugby employs stringent restrictions on the types of protective headgear allowed during gameplay [11]. The requirements for headgear approval were relaxed in the more recent Law 4 trial assessment [12], allowing the introduction of a new generation of headgear showing promising results for brain injury reduction in the laboratory [13–15]. Specifically, the limit on material density (previously limited to 45 kg/m^3^) and prohibition of sandwich construction were lifted. Despite this change, the testing methods and metrics used to evaluate impact attenuation remain largely unchanged.

The World Rugby standard requires headgear fitted to a steel headform conforming to EN960 [16] to impact a steel surface with an impact energy of 13.8 J. Peak linear accelerations (PLA) of the impacts must exceed 200 g. World Rugby introduced this attenuation limit to encourage players to protect themselves rather than use equipment that materially provides injury protection. The most recent World Rugby standard requires headgear, fitted to a steel headform conforming to EN960 [16], to be dropped onto a steel impact surface from heights of 15−60 cm with no clear criteria for interpreting the resulting impact kinematics [12]. Nearly all the laboratory studies of the impact attenuation of rugby headgear use a drop test method more closely aligned with the NOCSAE standard for American football helmet testing [17] thus, using either the Hybrid III or NOCSAE headform with either a rubber or fake turf impact surface [13, 15, 18–20]. The exception is Ganly et al. whose methodology to investigate their NPro headgear was more closely aligned with the World Rugby standard [11, 14].

Kinematic measures of head motion during impacts, such as PLA, peak rotational acceleration (PRA), and peak rotational velocity (PRV), are commonly reported to quantify brain injury risk due to both the relative ease of measurement, as accelerometers are affordable and widely available, and the view that kinematics offer an indication of the brain’s inertial response [21]. It has long been known that rotational kinematics alone elicit significant structural damage to the brains of primates [22–26], whilst several studies have found that linear kinematics alone did not correlate well with diagnosed brain injury on field [27–29]. A study investigating the combined effects of the linear and rotational kinematics on a finite element (FE) head model found that linear kinematics below 15 g became insignificant relative to the rotational as the duration of the rotational motion increased [30]. Kinematic measures alone, however, fail to adequately describe the complex interactions of head motion, cellular strain, and region-specific effects on the levels of strain developed within the brain. Despite this, current research on the impact mitigation of rugby headgear (including our previous investigations) only considers the reduction of linear and rotational accelerations [13, 15, 18–20].

To date, the most adopted predictors of human brain injury are the peak regional brain strain (specifically the maximal principal strain—MPS) and axonal strain [29, 31–33], along with the peak rotational velocity and acceleration (PRV and PRA) [28, 34]. Recent developments in finite element (FE) modelling and machine learning have made it possible to generate both peak strain values [35, 36] and 3D maps of voxel-wise brain strain [37, 38] resulting from an impact *instantly* instead of the several hours, and prohibitively large computational power, conventional FE simulations require. These instantaneous deep learning brain injury models achieved high accuracy estimations of whole brain MPS with a root mean squared error of 0.022 (2.2% strain) compared to the conventional FE simulation [36]. When extended to provide the spatial (voxel-wise) distribution of strain throughout the entire brain [37], the instantaneous deep learning brain injury model was considered “sufficiently accurate” for 97.1% of impacts within the kinematic range: $900 < PRA < 5100$ rad/s^2^ and $2 < PRV < {\text{ }}40$ rad/s. The advent of such models makes it feasible to reconsider the evaluation metrics used to quantify the head impact mitigation of headgear during laboratory studies.

Brain strain metrics are beginning to be used to quantify the impact mitigation offered by various headgear [39–42]. These studies, however, are limited to cycling and American football helmets. There have been no studies on the potential reduction in brain strain metrics offered by soft-shelled rugby headgear. This study, therefore, analysed the reduction in peak rotational velocity and peak regional brain strains offered by various rugby headgear during laboratory drop testing.

## Methods

### Headgear Choice

Five different models of branded headgear were used in this study which remain anonymous to limit the comparison to the materials involved and the headgear design. These headgear, therefore, are hereon labelled as headgear 1–5. Headgear 1–3 were conventional models of headgear which only claim to mitigate trauma to the ears and minor cuts and scrapes. Headgear 4 and 5 were chosen based on the manufacturer’s claims of impact acceleration mitigation during laboratory tests. All headgear was medium-sized as it best fit the circumference measurement of the headform across all headgear tested. Headgear 1 incorporates lightweight polyurethane foam $(\le 45)$ kg/m^3^ arranged into rectangular cells around the headgear. Headgear 2 uses lightweight $(\le 45)$ kg/m^3^ polyethylene foam formed into cells replicating the logo shape. Headgear 3 uses a lightweight $(\le 45)$ kg/m^3^ ethylene vinyl acetate (EVA) foam arranged in honeycomb-shaped cells. Headgear 4 uses a thicker, higher density $(\ge 45)$ kg/m^3^ open-cell polyurethane foam in square cells of varied size [14]. Headgear 5 uses EVA foam and a layer of impact-absorbing foam developed by D3O$^\circledR$ $(\ge 45)$ kg/m^3^ [43]. Headgears 4 and 5 use viscoelastic, open-celled foams compared to headgears 1, 2 and 3, which use closed-cell foams. Headgear 5 was the thickest unit (15–20 mm max thickness), compared to headgear 4 (12–13 mm max thickness) and headgear 1–3 (8–10 mm max thickness). Thickness was measured at the forehead, rear boss and side locations for all headgear, as these were the most easily accessible areas. All headgear fit tightly on the headform with no slippage, ensuring a consistent impact region throughout testing. This was crucial as the reliability of rotational data can be compromised if there is inappropriate coupling to the headform. All headgear was new and in unused condition. All impact locations were to padded areas of the headgear although motion of the head during impact may have resulted in a region of low padding experiencing the impact force. The effects of this were assumed to be negligible for this study, as the initial impact site was always on the padded areas.

### Data Collection

**Fig. 1:**
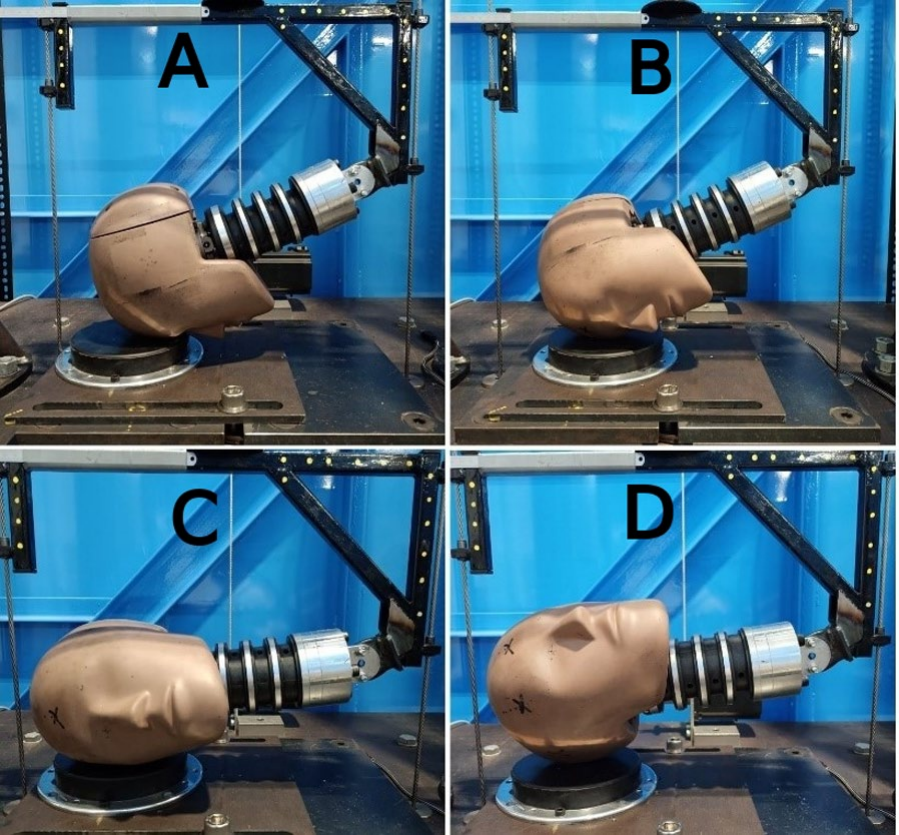
Impact locations onto the flat MEP pad [15]. From top left to bottom right: forehead (**A**), front boss (**B**), side (**C**), and rear-rear boss (**D**)

**Fig. 2:**
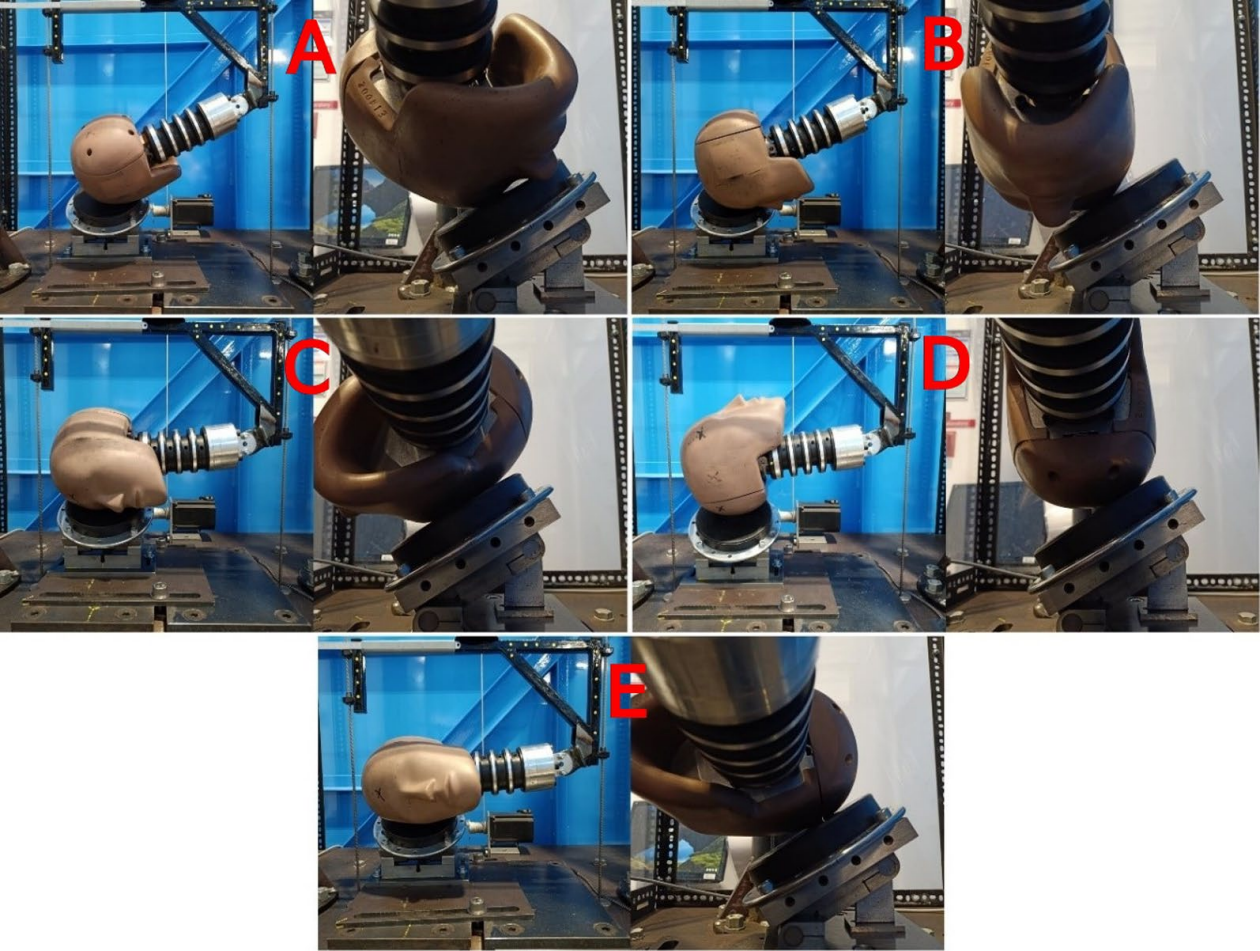
Impact locations onto the 45$^{\circ }$ impact surface [15]. Images are paired showing the angle of the head and neck and the point of impact on the head. From top left to bottom right: forehead (**A**), front boss (**B**), side (**C**), rear-rear boss (**D**), side-rear boss (**E**)

Impacts were carried out on a twin wire guided drop test rig using a Hybrid III (HIII) headform and neck instrumented with four triaxial accelerometers (Analog Devices ADXL377, 20,000 Hz, range: $\pm 200$ g, sensitivity: 6.5 mV/g) arranged into a nine accelerometer package (NAP) [44] with three redundant sensing axes. This allowed linear and rotational accelerations and rotational velocity to be measured and calculated. Drop test kinematic data was taken from previous work by Stitt et al. and Draper et al. comparing the potential of rugby headgear to reduce linear and rotational accelerations [15, 45]. These drop tests were carried out on a 1-inch modular elastomer pad (MEP) angled at 0 and 45$^{\circ }$ relative to the test rig base [15, 45]. All impacts were carried out across four impact locations: forehead, front boss, side, and rear boss (labelled rear-rear boss), as shown in Figs. 1 and 2. Impacts onto the $45^{\circ }$ surface also included a fifth impact location labelled side-rear boss (Fig. 2).

Four drop heights were chosen from the World Rugby Law 4 trial assessment (15, 30, 45, and 60 cm) (Table 1). Heights above 60 cm were likely to damage the testing equipment, head, and neck and were therefore avoided. Impacts were repeated five times with 60 s between each consecutive impact. New headgear was used after 20 drop tests in each location to mitigate the effect of material degradation. Since it is unknown how much of the total falling mass is involved in the acceleration peak, impact energies in Table 1 were determined for the head and neck only (5.6 kg), and the total falling mass of 6.8 kg including the drop frame. In total there were 480 drop tests carried out onto a flat impact surface, and 600 carried out onto the 45$^{\circ }$ impact surface. The disparity was due to the extra impact location incorporated into the 45$^{\circ }$ impact surface drop tests.

**Table 1:** Drop heights used in this study with the equivalent impact velocity and impact energies

Drop height (cm)	Impact velocity (m/s)	Head/neck impact energy (J)	Whole system impact energy (J)
15	1.7	8.2	9.8
30	2.4	16.1	19.6
45	3.0	25.2	30.6
60	3.4	32.4	39.3

### Brain Strain Estimation

**Fig. 3:**
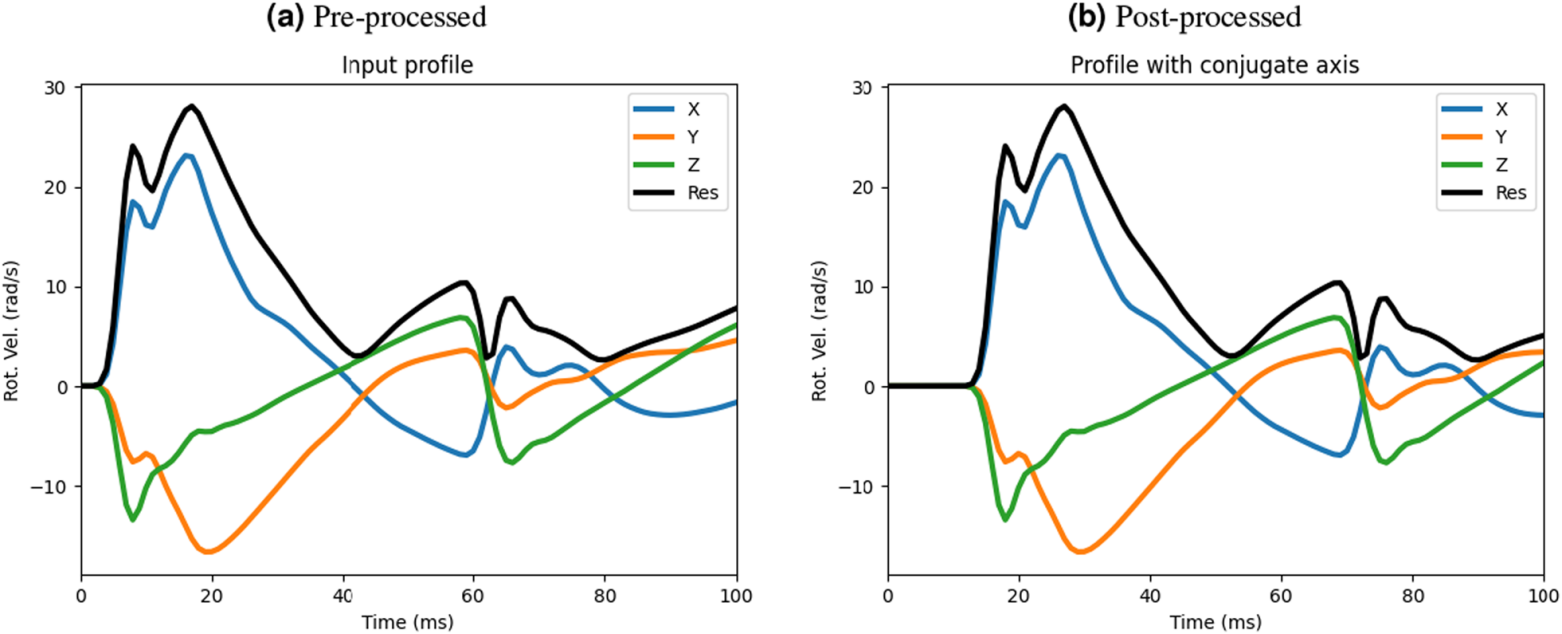
Rotational velocity trace for a rear, rear boss impact onto the 0$^{\circ }$ impact surface showing **a** the pre-processed rotational velocity trace, and **b** the rotational velocity trace following the preprocessing steps in the publicly available pre-trained CNN model

A publicly available pre-trained convolutional neural network (CNN) model was used as an instantaneous deep learning brain injury model to estimate 3D voxel-wise maximal principal strain (MPS) over the entire brain in each head impact [35–37]. The model was trained on the Worcester Head Injury Model (WHIM v1.0) [46] and was deemed “sufficiently accurate” compared to the peak MPS of the conventionally simulated counterpart for 97.1% of impacts within the kinematic range: $900 < PRA < 51002$ rad/s^2^ and $2 < PRV < 40$ rad/s [37]. The range of peak rotational velocity and acceleration measured by the headform sensors for this study was 7.6–38 rad/s (25th and 75th percentile of 14.4 and 23.1 rad/s) and 890–7345 rad/s^2^ (25th and 75th percentile of 2371 and 4461 rad/s^2^). It should be noted that two separate CNN models reside within the provided GitHub repository. The first estimates only the peak MPS of the corpus callosum and whole brain, while the second more recent model (used in this study) provides the spatial distribution of the peak MPS across the entire brain. Since this model only accepted data recorded at 1000 Hz, time-series head impact data was resampled to achieve the desired sampling rate. Linear kinematics were significantly altered in shape and peak value during resampling. Fortunately, the CNN model only uses rotational velocity and acceleration to make predictions of the voxel-wise strain [28], neither of which were affected by the resampling. Time series rotational velocity data was preprocessed using the respective codes published in the repository, thus ensuring the azimuth and elevation of rotational velocity axes matched that required for the CNN model. Figure 3 shows an example rotational velocity time-series trace before and after pre-processing for a rear, rear boss impact. This model has also been used to investigate the impact mitigation of American football helmets [42]. Peak maximal principal strain (MPS) values were extracted from the corpus callosum, brainstem, and cerebral hemispheres while cumulative strain damage measures (CSDM$_{15}$) were extracted from the corpus callosum and the brainstem only. CSDM$_{15}$ was calculated as the fraction of voxels with a peak MPS greater than 15% strain.

The coordinates of the voxels for the corpus callosum were provided by the developers of the CNN model. The brainstem voxel locations were estimated with the help of local field experts. The brainstem voxels began directly under the corpus callosum and included the regions of the midbrain, pons, and medulla. The cerebrum included the remaining voxels of the brain, excluding the cerebellum. The cerebrum, therefore, included the temporal, frontal, parietal, and occipital lobes. While the instantaneous deep learning brain injury model does not inform the temporal history of the regional brain strains, research by Ji et al. has shown the MPS in the deep brain regions to peak within about 20 ms of the rotational velocity peak [47]. The data acquisition window for each impact in this study (about 10 ms pre impact, and 110 ms post impact) was assumed to be long enough to capture the maximum MPS values reached from each drop test.

### Statistical Analysis

For comparison between headgear and no-headgear cases, impacts were grouped across all impact locations and compared at different drop heights and impact surface angles. The distribution of peak rotational velocities and regional brain strains across the impact locations did not follow a known distribution, therefore, bootstrapping, with 10,000 repeats, was used to construct 95% confidence intervals of the mean and the difference between the means of each headgear and the no headgear case. Bootstrapping was carried out using python’s “bootstrap” function from the “scipy” library. Significance testing was only carried out between each headgear and the no-headgear case for each impact surface angle and drop height. Statistical tests were not carried out between the headgear types. Results are presented as the 95% distribution of the means resulting from bootstrapping with the median value explicitly stated.

## Results

**Fig. 4:**
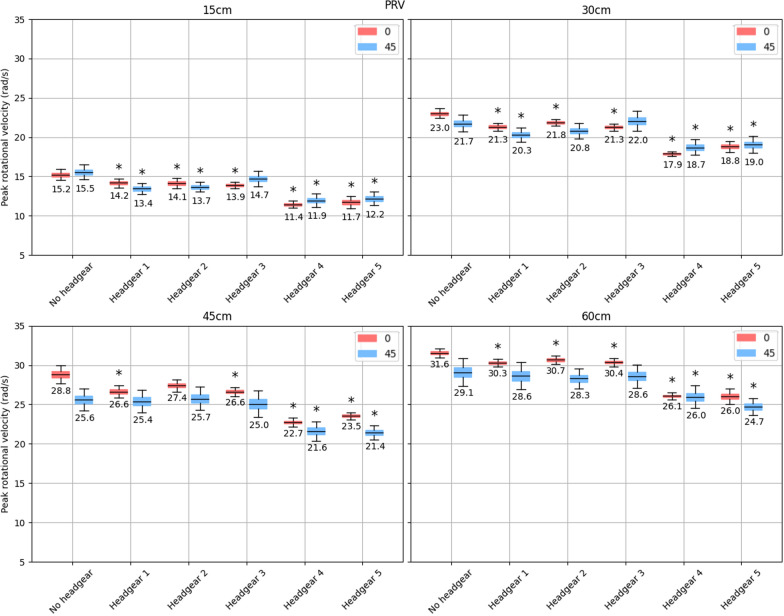
95% bootstrapped confidence intervals for the mean peak rotational velocity (with the central value shown) across all impact locations for each headgear at drop heights of 15, 30, 45, and 60 cm (top left, top right, bottom left, and bottom right respectively). The red shows impacts onto the flat surface, while the blue shows impacts onto the 45$^{\circ }$ surface. Stars indicate a significant increase or decrease in PRV compared to the no-headgear case for the same impact surface angle

All headgear showed reductions in PRV, but this reduction did not always reach statistical significance (Fig. 4). At 15 cm drop heights, headgear 1 and 2 significantly reduced PRV on the 0 and 45$^{\circ }$ impact surfaces, while headgear 3 only made a significant reduction onto the flat impact surface and not the angled surface. Similarly, on the 30 and 60 cm impacts, headgear 1–3 provided statistically significant reductions in PRV on the flat impact surfaces with only headgear 1 significantly reducing the PRV at 30 cm on the angled surface. On the 45 cm impacts, only headgear 1 and 3 significantly reduced PRV compared to no headgear and only on the flat impact surface. Across all drop heights and impact surface angles, headgear 4 and 5 significantly reduced the peak rotational velocities compared to no headgear. Across all drop heights and impact surface angles, the maximum reduction in median PRV was between 4.3 and 6.1rad/s and was seen on the flat impact surface by headgear 4 and 5. A list of the 95% confidence intervals for the means and the difference in means between each headgear and the no headgear case can be found in the supplementary file.

**Fig. 5:**
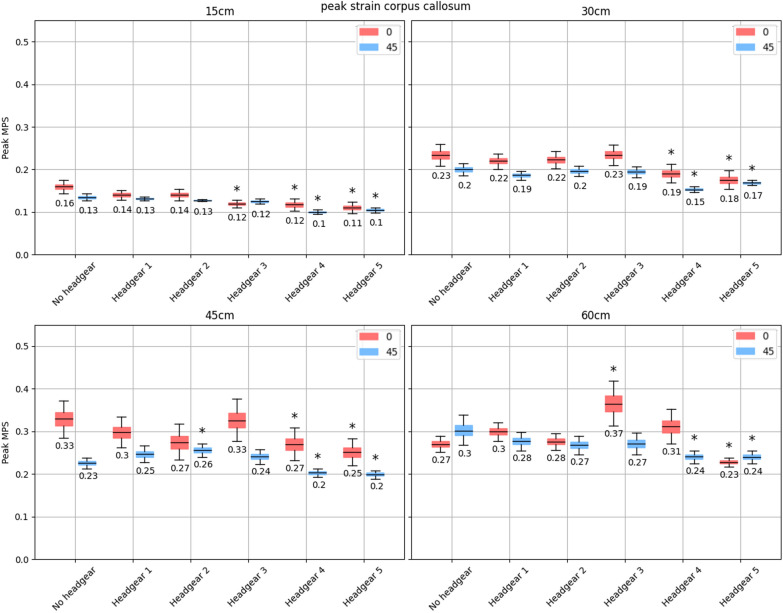
95% bootstrapped confidence intervals for the mean peak MPS in the corpus callosum (with the central value shown) across all impact locations for each headgear at drop heights of 15, 30, 45, and 60 cm (top left, top right, bottom left, and bottom right respectively). The red shows impacts onto the flat surface, while the blue shows impacts onto the 45$^{\circ }$ surface. Stars indicate a significant increase or decrease in peak MPS compared to the no-headgear case for the same impact surface angle

Unlike the PRV, most headgear units failed to reduce peak corpus callosum strain to a statistically significant extent (Fig. 5). Peak strain in the corpus callosum was lowered to a statistically significant level by headgear 4 and 5 across all four drop heights, except at the 60 cm drop height on a flat impact surface where headgear 4 displayed a non-significant increase in the regional peak strain. At 15 cm drop heights, headgear 4 and 5 displayed a decrease in the mean peak corpus callosum strain of [0.02, 0.06] and [0.03, 0.07] from the no headgear case during impacts onto the flat and [0.02, 0.04] and [0.02, 0.04] on the 45$^{\circ }$ impact surfaces respectively. At 30 cm drop heights, headgear 4 reduced peak MPS values by [0.01, 0.08] on the flat and [0.03, 0.06] on the 45$^{\circ }$ impact surfaces. Similarly, headgear 5 reduced peak MPS by [0.02, 0.09] and [0.02, 0.05] on the flat and angled impact surfaces. A similar reduction occurred at the 45 cm drop heights. An exception was at the 60 cm drop height onto a flat impact surface where headgear 4 increased the peak MPS (95% CI [− 0.09, 0.005]) in the corpus callosum compared to the no headgear case. This result, however, did not reach statistical significance. All other headgear rarely reduced the peak MPS in this brain region to a significant extent, with only headgear 3 showing a statistically significant reduction in peak regional MPS at the 15 cm drop height onto the flat impact surface. Headgear 2 significantly increased peak MPS during angled impacts at 45 cm (95% CI of MPS increase [0.01, 0.05]), while headgear 3 significantly increased peak corpus callosum MPS during 60 cm impacts onto the flat surface (95% CI of MPS increase [0.04, 0.15]).

**Fig. 6:**
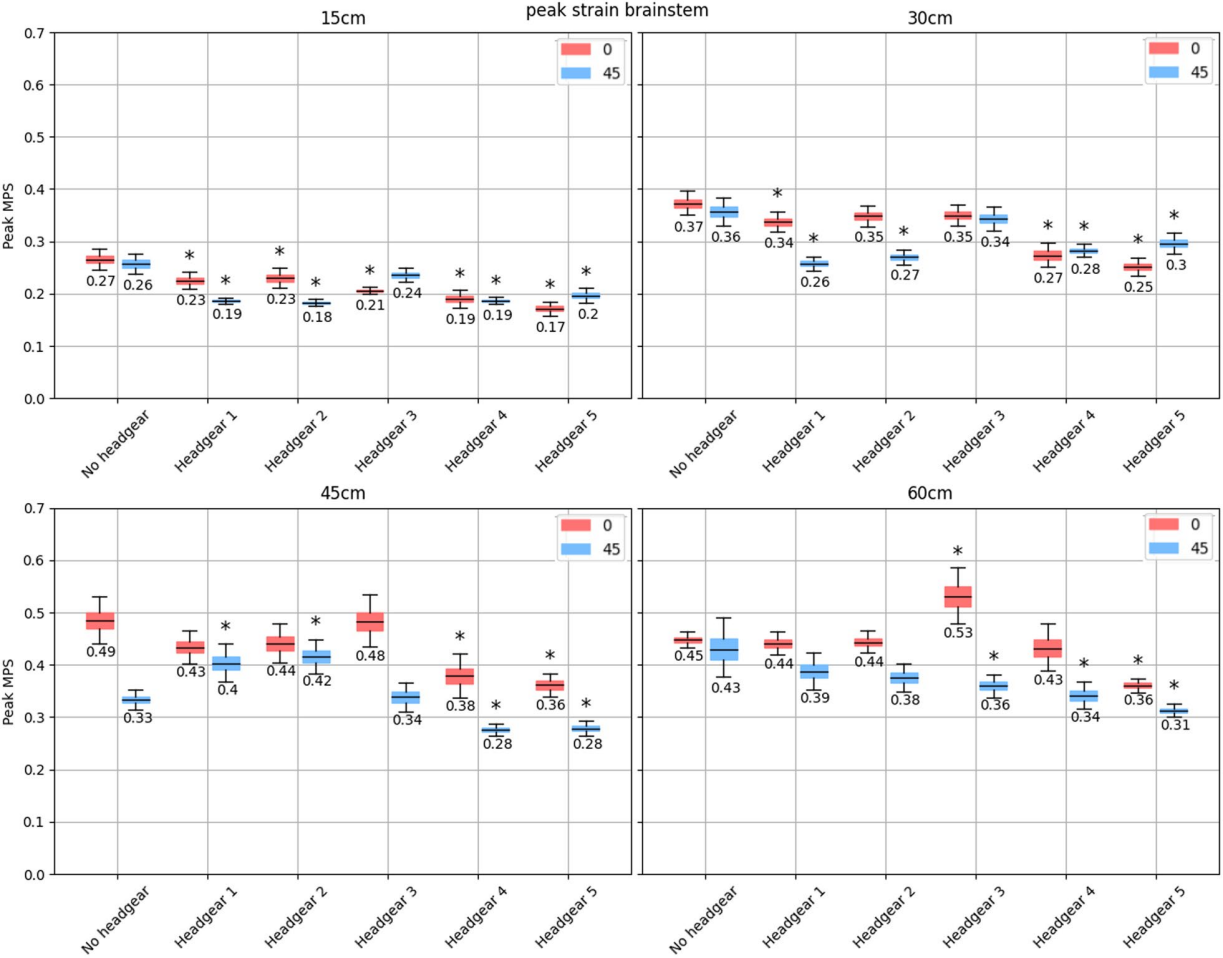
95% bootstrapped confidence intervals for the mean peak MPS in the brainstem (with the central value shown) across all impact locations for each headgear at drop heights of 15, 30, 45, and 60 cm (top left, top right, bottom left, and bottom right respectively). The red shows impacts onto the flat surface, while the blue shows impacts onto the 45$^{\circ }$ surface. Stars indicate a significant increase or decrease in peak MPS compared to the no-headgear case for the same impact surface angle

In line with the corpus callosum, headgear 4 and 5 significantly reduced the peak MPS in the brainstem across nearly all drop heights and impact surface angles (Fig. 6). The exception was at the 60 cm drop height, where headgear 4 did not significantly lower the peak MPS in the brainstem. The reductions in peak MPS offered by headgear 4 and 5 were also larger than those of the corpus callosum across the drop heights. Headgear 1 and 2 significantly reduced peak brainstem MPS at 15 cm drop heights across both impact surface angles but did not consistently lower peak MPS in the brainstem at increased drop heights. Headgear 3 only reduced peak MPS at 15 cm and 60 cm drop heights and only on the flat and angled impact surface respectively, while resulting in a significant increase in the peak MPS during flat impacts at the 60 cm drop height (95% CI of MPS increase [0.03, 0.14]). Headgear 1 and 2 also showed a significant increase in the peak brainstem MPS during 45 cm drop heights onto an angled impact surface (95% CI of MPS increase [0.03, 0.11] and [0.04, 0.12] respectively).

**Fig. 7:**
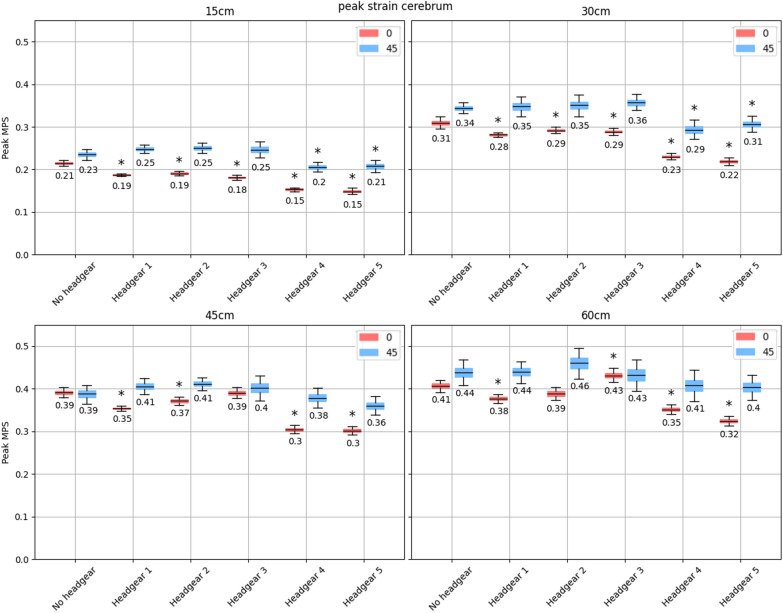
95% bootstrapped confidence intervals for the mean peak MPS in the cerebral hemispheres (with the central value shown) across all impact locations for each headgear at drop heights of 15, 30, 45, and 60 cm (top left, top right, bottom left, and bottom right respectively). The red shows impacts onto the flat surface, while the blue shows impacts onto the 45$^{\circ }$ surface. Stars indicate a significant increase or decrease in peak MPS compared to the no-headgear case for the same impact surface angle

Unlike the corpus callosum and the brainstem, consistently higher peak strains were observed in the cerebral hemispheres during angled impacts than flat impacts in all headgear cases across all four drop heights (Fig. 7). At 15 cm drop heights, all headgear significantly reduced the peak MPS during impacts onto the flat impact surface. On the 45$^{\circ }$ impact surface, at the same height, only headgear 4 and 5 reduced the peak MPS to a significant extent. Headgear 4 and 5 also significantly reduced peak MPS on both impact surface angles at the 30 cm drop height. This trend did not continue at increased drop heights as headgear 4 and 5 only showed a significant reduction in the peak cerebral MPS on flat impact surfaces at the 45 and 60 cm drop heights. Interestingly, headgear 1 significantly reduced peak cerebral MPS at all drop heights but only onto the flat impact surface. Headgear 2 and 3 significantly reduced the cerebral MPS at 15 and 30 cm drop heights onto a flat impact surface, but only headgear 2 continued this reduction at the 45 cm drop height. Between 15 and 45 cm drop heights, headgear 1–3 showed an increase in the peak cerebral MPS during impacts onto the 45$^{\circ }$ surface, however, these increases did not reach significance. During 60 cm drop heights, headgear 1 and 2 continued the non-significant increase in MPS while headgear 3 displayed the same significant increase in peak MPS during flat impacts as was observed in the corpus callosum and brainstem (95% CI of MPS increase [0.002, 0.05]).

Appendix Figs. 9 and 10 show the CSDM$_{15}$ for the corpus callosum and the brainstem across the drop heights and impact surface angles tested. CSDM$_{15}$ was only significantly reduced by all headgear during 15 cm impacts in the corpus callosum. Above this height, only headgear 4 and 5 significantly reduced the CSDM$_{15}$ in this brain region. At 60 cm drop heights, headgear 4 and 5 only significantly reduced the CSDM$_{15}$ in this region during flat impacts and not during impacts onto the angled impact surface angle. Similarly, all headgear reduced the CSDM$_{15}$ to a significant extent in the brainstem during 15 cm impacts, both onto the flat and angled impact surfaces. At the 30 cm drop height only headgear 1, 4, and 5 significantly reduced CSDM$_{15}$ across both impact surface angles with headgear 2 only providing a significant reduction during angled impacts, and headgear 3 not showing any significant reduction. At 45 cm drop heights, headgear 1–3 all significantly reduced the CSDM$_{15}$ in the brainstem during flat impacts compared to no headgear, however, at the 60 cm drop height, only headgear 1 and 3 reduced the CSDM$_{15}$ in this region during impacts onto the flat and 45$^{\circ }$ surface respectively. Headgear 4 and 5 significantly reduced the CSDM$_{15}$ in the brainstem across all 4 drop heights and both impact surface angles.

## Discussion

Unlike the previous investigations of the impact mitigation of rugby headgear where all headgear significantly reduced the peak linear and rotational accelerations [13, 15], the present study, when considering metrics more strongly associated with diagnosed concussive injury, did not find such consistent results. The relationship between brain strain and head kinematics is complex. Measuring the head kinematics alone may be insufficient to understand the effect of headgear on the true metric of interest: brain injury risk. The results presented here corroborate this by showing relatively unclear and inconsistent trends in brain strain reduction across different headgear and impact intensities despite the conclusive nature of our previous studies. Peak rotational velocities were generally lower when headgear was present on the flat impact surface, but this reduction was not consistently significant with headgear 1–3. In some isolated cases, an increase in the peak rotational velocity was found, however, these increases did not reach statistical significance. Similarly, the same three headgear units rarely lowered the peak regional MPS while increasing it in some cases to a statistically significant extent. In contrast, headgear 4 and 5 generally achieved significantly lower regional brain strains than the no headgear case. These reductions, however, were small in the corpus callosum. Generally, headgear presence appeared to have a more pronounced effect on the peak strain in the brainstem. This difference was most notable with the headgear 4 and 5, reducing the mean peak MPS by 0.12–0.13 (12–13% strain) compared to the no headgear case. At the highest intensity impacts (60 cm), however, the headgear generally achieved lower absolute reductions in peak brainstem strain. This trend was also observed in the cerebrum, with lower reductions in peak MPS at 60 cm drop heights than at lower drop heights.

The similarity in impact attenuation behaviour between headgear 1–3 and headgear 4 and 5 is explainable via the mechanical properties of the foams used. These first three headgear incorporate lightweight ($\le 45$ kg/m^3^) closed cell foam at a thickness of 8–10 mm in similar cell structure arrangements around the headgear. Headgear 4 comprises a higher density, viscoelastic, open cell polyurethane foam [48], and headgear 5 uses a layer of EVA foam and a layer of higher density, impact absorbing, viscoelastic foam developed by D3O$^\circledR$ [43]. Closed-cell foams comprise many tiny pockets of air trapped within cells made of the foam polymer. Energy is primarily absorbed through compression of the internal air pockets and deformation of the cell walls, giving the foams their springy feel when compressed [49–51]. Open-cell foam cells are not fully closed off, allowing air to move through the material. Energy absorption occurs through the deformation of the polymer structure, which forces air through the cellular structure. The viscous forces created by air moving through the foam also dissipate a significant amount of energy [49, 51, 52]. At equivalent densities, open-cell foams are far more compliant than closed-cell foams [53]. High density open-cell foams, therefore, are better suited for soft-shelled headgear than high density closed-cell foams. A higher density foam incorporates a greater proportion of the solid polymer relative to the amount of air trapped inside the foam. The higher proportion of the polymer in the open-cell foams may allow for a greater dissipation of the kinetic energy during impact through polymer deformation. This increased foam density and viscoelastic response of the open cell foams, compared to the closed-cell foams, likely account for most of the difference in impact attenuation behaviour between the two foam types.

Additionally, headgear 4 and 5 exhibited a noticeable memory foam effect when deformed, likely due to the glass transition temperature of the foams being near that of the laboratory temperature. This property allows significant deformation of the foam structure without permanent damage [54] and allows the foam to restore its original shape after some time. While likely contributing to the impact mitigation observed in the present study, a significant change may occur in impact mitigation properties with increased temperature. Higher temperatures were excluded from this study but would be necessary to better understand the behaviour of headgear during a gameplay scenario, where temperatures inside the headgear are increased [55]. When close to the glass transition temperature, a change of just a few degrees in either direction could significantly affect the mechanical properties of a foam.

**Fig. 8:**
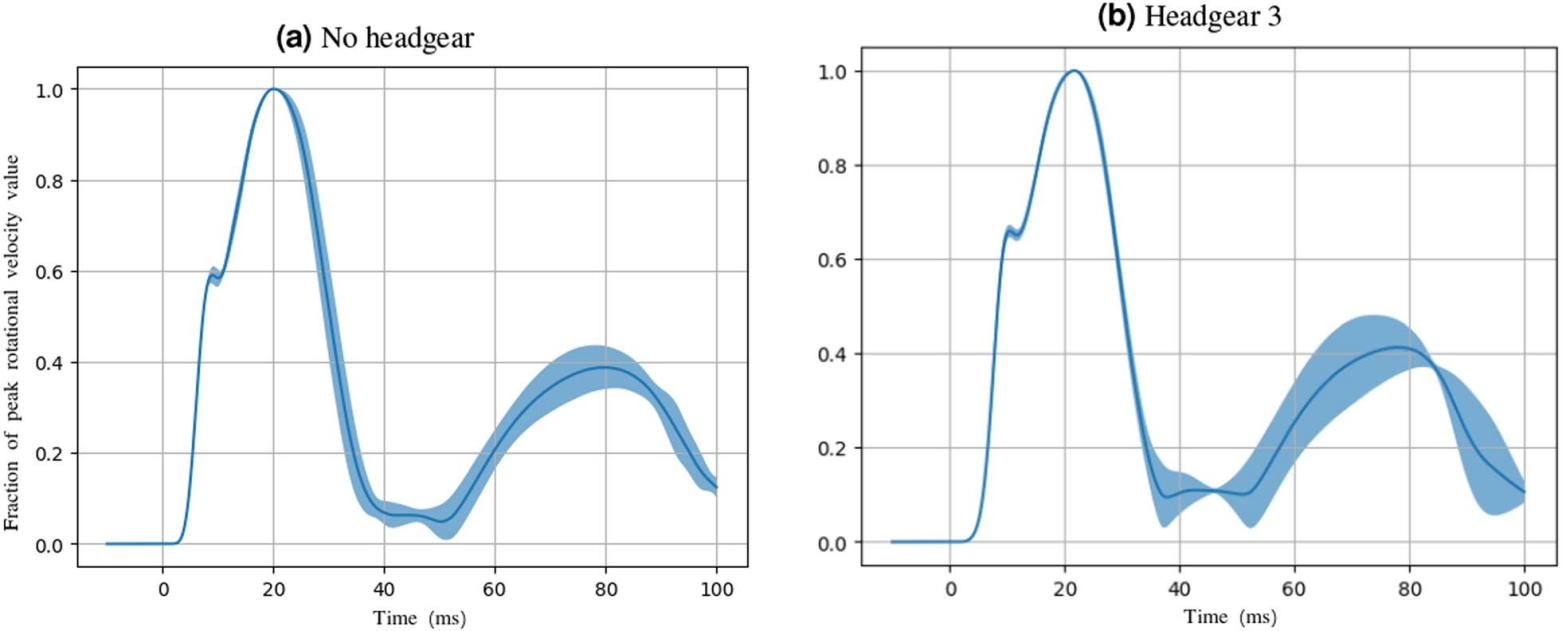
Mean and standard deviations of the normalised resultant rotational velocity traces for forehead impacts with no headgear and headgear 3 illustrating the slight difference in post-peak kinematics

The material properties may also explain the slight increase in the brain strains from the no headgear case seen periodically with the use of headgear 1–3. The springy closed cell foams used in headgear 1–3, while lowering the peak linear and rotational accelerations, may increase the post-impact rebound due to the reforming of the closed cells acting as a spring. While this does not affect the peak value of the accelerations since they peak when the headform, headgear, and impact surface have reached maximum compression, the transfer of energy into the deformation of the brain structure likely has some dependence on post-peak kinematics. These post-peak kinematics may be affected by the presence of certain headgear types. Figure 8 illustrates this using headgear 3 forehead impacts as an example. Following the peak rotational velocity, the resultant trace for headgear 3 shows a slightly higher average secondary peak and a larger associated standard deviation than the no headgear case. While these differences are small, they may account for the increased brain strain values observed during drop testing. Additionally, these foams may have a high resistance to shear loading, as would likely be seen during angled impacts. Headgear may also slightly increase the distance between the point of impact and the centre of mass of the headform. Along with high resistance to shear deformation, this increase may affect the peak rotational velocity reached for the same impact force. The results of this study suggest open cell foams may absorb more impact energy through this type of deformation despite the potential increase in distance between the impact point and head centre of mass due to the increased headgear thickness.

Regarding ideal headgear behaviour, Post et al. have investigated how the linear acceleration curves affect the peak MPS when simulating the impact on the University College Dublin Brain Trauma Model [56]. Three separate acceleration curves were developed representing an impact mitigated by a soft material that “bottoms out”, a dense material that absorbs minimal impact energy (B), and an idealised material resulting in a “plateau curve” with the lowest peak acceleration. Impacts with the soft material consistently produced the highest stresses and strains while the idealised acceleration curve produced the lowest, except for two cases where the dense material showed the lowest. This study suggests that there may be an idealised headgear response for minimising the brain strain induced by a head impact and that either a dense material or a material with a large capacity to absorb energy should be used. Since this study was limited to the link between acceleration loading and brain strain, it is not known how the changes in the shape of the rotational velocity curve would be affected by the material properties of a head impact mitigation material.

One major limitation of this study is the lack of understanding of how well the laboratory drop test conditions employed in this study reflect those experienced by rugby players during gameplay. Unfortunately, there is minimal research describing the relationship of laboratory impact testing to the impact conditions that exist during rugby gameplay. There has, however, been a significant amount of research investigating the link between head impact conditions in ice hockey and those recreated in the laboratory and the associated impact attenuation of ice hockey headgear under these conditions. In 2016 and 2018, Clark and colleagues measured the impact attenuation of standard ice hockey helmets for different impact events in ice hockey [57, 58]. Using a HIII head and an unbiased neckform, the authors carried out drop tests onto an MEP pad (representing falls onto ice), linear impact tests with a nylon hemispherical pad and a 36 mm thick vinyl nitrile pad underneath (representing elbow strikes), and a (68 mm) vinyl nitrile foam pad and a reebok shoulder pad (recreating shoulder strikes). Linear and rotational accelerations, along with finite element simulated peak MPS in the cerebrum, were used to quantify the impact attenuation of the helmets. The authors found that the helmets had a diminishing effect on impact duration, peak accelerations, and peak MPS during impacts onto a more compliant surface (shoulder impacts) than during impacts with a less compliant surface (falls, elbow collisions, and puck impacts) compared to unhelmeted conditions. In both studies, the authors reported this to arise from the materials in the ice hockey helmets being stiffer than those of the impact surface. As a result, the helmet materials do not compress enough to absorb the impact energy, thereby minimizing impact attenuation. A study by de Grau and colleagues [59] also found that the reductions in peak kinematics and brain strain through the use of an ice hockey helmet were higher during low compliance impacts compared to high compliance impact conditions. A further study by Haid and colleagues carried out 1 m high free-fall drop tests with a HIII headform onto surfaces with varying compliance (1-inch MEP pad and layered EVA foam sheets giving 24, 48, 72, and 96 mm overall thickness), comparing the impact attenuation of ice hockey helmets across the different surfaces [60]. When increasing impact surface compliance, the difference between helmeted and unhelmeted impacts decreased until a fitted helmet made no measurable difference to the peak accelerations. While there are substantial differences between the materials used to construct ice hockey and soft-shelled rugby headgear, the results of these studies likely hold relevance for future investigations of rugby headgear. The MEP pad used in the present study may only reflect a single, potentially uncommon impact condition where headgear could significantly attenuate head impact and brain strain metrics. Testing in such a way may provide an inflated indication of the potential of rugby headgear to reduce the risk of brain injury during rugby gameplay.

The instantaneous deep learning brain injury model does not include all brain structures (e.g. sulci, falx), although the training model (WHIM v1.0) incorporates these features and white matter anisotropy. The output of the instantaneous deep learning brain injury model does not inform the temporal history of the strain throughout the brain. While there is an extension of the CNN model used in this study that offers the spatiotemporal brain-skull relative displacement resulting from impact [61], this model does not appear to be publicly available for use. This analysis may be important for a deeper understanding of the relationship between mitigation of peak values during impact and the changes in temporal strain history. Some impacts, while having peak rotational velocities within the range considered accurate by the developers of the CNN model, had peak rotational accelerations that were outside the range deemed accurate. Although the results suggest newer generation headgear may be able to lower brain strain during head impacts, a well-validated drop test methodology based on rugby-specific head impact is crucial to understanding their real-life impact mitigation, along with quantification through a more robust finite element model.

This study aimed to further our previous investigations of soft-shelled rugby headgear [13, 15] by quantifying the impact mitigation via metrics more strongly associated with diagnosed concussive injury than linear and rotational accelerations alone. These metrics included the peak regional brain strain and the peak resultant rotational velocities. The methods employed in the original study of linear and rotational acceleration mitigation of rugby headgear had several limitations. A matching impact location for the side-rear boss impacts onto the flat was not found. When attempting to recreate the side-rear boss position on the flat impact surface, the headform easily and consistently contacted the surface on which the MEP pad was mounted. Ultimately it was not deemed possible to represent this impact location on a flat impact surface with the equipment available. Secondly, the impact conditions created in this study may not accurately reflect real-life, rugby-specific head impacts. Unfortunately, a detailed comparison between rugby head impacts and head impact simulations in the laboratory is lacking. In addition, the HIII headform used here has limited biofidelity and only partially represents head geometry and possible head impact response of a rugby player cohort. The HIII headform is, however, widely accepted and used in head impact research.

## Conclusions

This study aimed to evaluate the efficacy of soft-shelled rugby headgear in mitigating head impact metrics associated with diagnosed concussive injuries in a laboratory setting. Headgear effectiveness depended on the materials used and their interaction with the various impact conditions. Notably, headgear constructed with lightweight, closed-cell foam showed varied PRV and MPS reduction across different drop heights and impact surface angles. In some cases, they significantly lowered these metrics, while in most others, the mitigation was not statistically significant. In contrast, headgear 4 and 5, which employed higher-density, viscoelastic, open-cell foam, consistently demonstrated significant reductions in PRV and MPS. However, the use of the MEP pad may not adequately reflect the specific impact conditions experienced during rugby gameplay. This underscores the need for a well-validated drop test methodology tailored to rugby-specific head impacts. Such an approach could provide a more accurate representation of real-world scenarios and enhance our understanding of the true impact mitigation capacity of rugby headgear.

## Supplementary Information

Supplementary Material 1.

## Figures and Tables

**Fig. 9:**
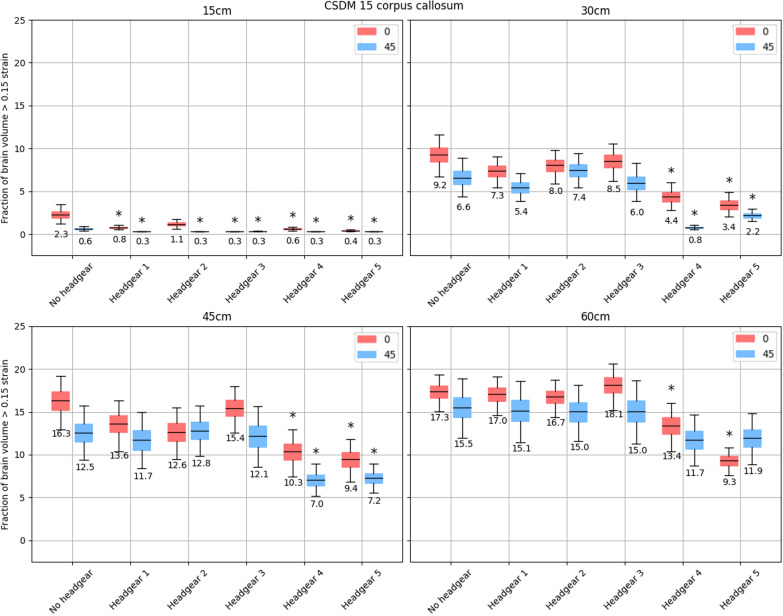
95% bootstrapped confidence intervals for the mean CSDM$_{15}$ in the corpus callosum (with the central value shown) across all impact locations for each headgear at drop heights of 15, 30, 45, and 60 cm (top left, top right, bottom left, and bottom right respectively). The red shows impacts onto the flat surface, while the blue shows impacts onto the 45$^{\circ }$ surface. Stars indicate a significant increase or decrease in CSDM$_{15}$ compared to the no-headgear case for the same impact surface angle

**Fig. 10:**
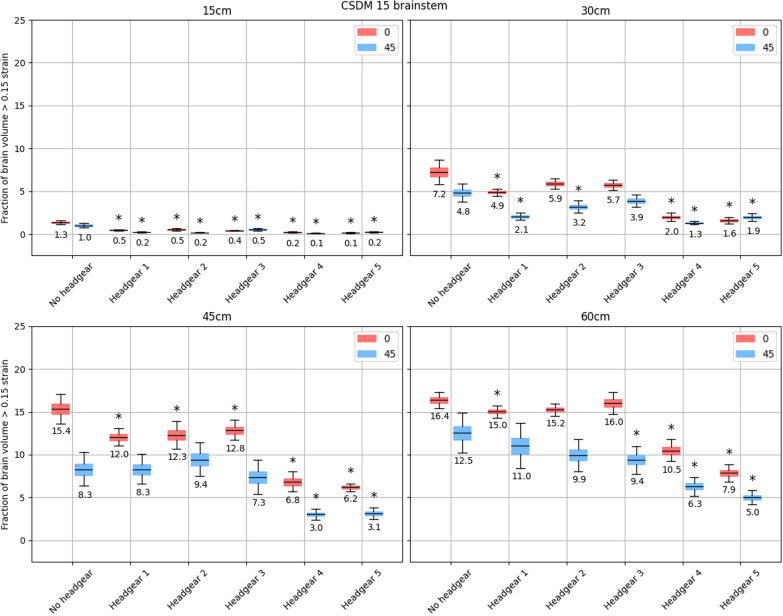
95% bootstrapped confidence intervals for the mean CSDM$_{15}$ in the brainstem (with the central value shown) across all impact locations for each headgear at drop heights of 15, 30, 45, and 60 cm (top left, top right, bottom left, and bottom right respectively). The red shows impacts onto the flat surface, while the blue shows impacts onto the 45$^{\circ }$ surface. Stars indicate a significant increase or decrease in CSDM$_{15}$ compared to the no-headgear case for the same impact surface angle

## Data Availability

The datasets used and/or analysed during the current study can be made available from the corresponding author upon reasonable request.

## Appendix

See Figs. 9 and 10.
