# Supplementary material for: Potential of Soft-Shelled Rugby Headgear to Lower Regional Brain Strain Metrics During Standard Drop Tests
Source: Sports Med Open. 2024 Sep 27;10:102. doi: 10.1186/s40798-024-00744-2 (PMC11436562; doi:10.1186/s40798-024-00744-2)
Supplement: Supplementary file 1 — Supplementary Material 1. [file 40798_2024_744_MOESM1_ESM.pdf]

Potential of soft-shelled rugby headgear to lower regional brain strain metrics during standard drop tests

Danyon Stitt[1,2], Natalia Kabaliuk[1,2,*], Keith Alexander[1], and Nick Draper[2,3]

[1]University of Canterbury, Department of Mechanical Engineering, Christchurch, 8041, New Zealand
[2]University of Canterbury, Sports Health and Rehabilitation Research Center (SHARRC), Christchurch, 8041, New Zealand
[3]University of Canterbury, Faculty of Health, Christchurch, 8041, New Zealand [*]natalia.kabaliuk@canterbury.ac.nz

Submitted to Sports Medicine - open

The following list shows the 95% confidence intervals for the mean peak kinematic and brain strain metric for each drop test condition without headgear. The list also shows the 95% confidence interval for the difference in means between each headgear and the no headgear case for each drop test condition.

PRV 15cm 0 no headgear
Mean (15.2180571655134, [14.515339896100006, 15.892228985800005])
PRV 15cm 0 ccc
Mean (14.178382283893638, [13.626332982624996, 14.789737849549995]) Difference [0.10195388160001029, 1.9320734022250072]
PRV 15cm 0 gilbert
Mean (14.135321126548655, [13.515649198125002, 14.772436994250002]) Difference [0.11900100287500504, 2.0172910838750013]
PRV 15cm 0 2nd skull
Mean (13.886605613646664, [13.500031870524998, 14.290389864499998]) Difference [0.5140784029750067, 2.1207023325000063]
PRV 15cm 0 npro
Mean (11.449056854331092, [10.973309499730004, 11.924878688695005]) Difference [2.907785176974996, 4.621692126975001]
PRV 15cm 0 gbreaker
Mean (11.707930718429369, [10.9323286901175, 12.499828665402498]) Difference [2.430379576137507, 4.563458500815004]
PRV 15cm 45 no headgear
Mean (15.54211229531157, [14.55745884996, 16.503316134459997])
PRV 15cm 45 ccc
Mean (13.434097665993601, [12.715179715900002, 14.125848304800005]) Difference [0.8965401206199957,

3.2893599203799937]
PRV 15cm 45 gilbert
Mean (13.654302387965998, [13.03043976962, 14.2785096646])
Difference [0.724706110559997, 3.0391602653000005]
PRV 15cm 45 2nd skull
Mean (14.71283720168297, [13.742890767519999, 15.6717442117])
Difference [-0.5558610568400053, 2.188897274879999]
PRV 15cm 45 npro
Mean (11.93558869470387, [11.086578594444, 12.759913039126001])
Difference [2.3055798376599963, 4.877898219675998]
PRV 15cm 45 gbreaker
Mean (12.18119842841657, [11.286354333180006, 13.020832041864])
Difference [2.061091501900001, 4.672746500528]

PRV 30cm 0 no headgear
Mean (22.97739061850875, [22.328765911625005,
23.551998837750002]) PRV 30cm 0 ccc
Mean (21.29136615958736, [20.802768621524997,
21.828897377924996]) Difference [0.8624136319750026,
2.484521471925003]
PRV 30cm 0 gilbert
Mean (21.847266605750377, [21.4206119756, 22.288261479499994])
Difference [0.36807405367500445, 1.885313170974999]
PRV 30cm 0 2nd skull
Mean (21.255585841341535, [20.813774209874992,
21.724695240424992]) Difference [0.9336298026000072,
2.4827958335750107]
PRV 30cm 0 npro
Mean (17.878485158210474, [17.600023604899995,
18.188117868349998]) Difference [4.397466937624999,
5.761582436050003]
PRV 30cm 0 gbreaker
Mean (18.783412778568504, [18.069857974350008,
19.513171932700008]) Difference [3.2302464411249954,
5.138422570425]
PRV 30cm 45 no headgear
Mean (21.73347864731341, [20.596890346899993,
22.811105919199992]) PRV 30cm 45 ccc
Mean (20.273744427458986, [19.393596899900004,
21.150923664620006]) Difference [0.020874586959992225,
2.8578637280599897] PRV 30cm 45 gilbert
Mean (20.774266237433746, [19.780142689820007,
21.751269359900007]) Difference [-0.5667708054200205,
2.4341875051599846] PRV 30cm 45 2nd skull
Mean (22.020684243683128, [20.736887442180006,
23.256094590920004]) Difference [-1.9748283841400112,
1.3816032415599941] PRV 30cm 45 npro
Mean (18.688582020390875, [17.642539655939995,
19.675154277359997]) Difference [1.5395241771199926,
4.55615464285999]
PRV 30cm 45 gbreaker
Mean (19.03399914961228, [17.951203683780008,
20.099293324660007]) Difference [1.1416254134799884,
4.264853236659989]

PRV 45cm 0 no headgear
Mean (28.802732473340136, [27.643827375199994, 29.949828020824985]) PRV 45cm 0 ccc
Mean (26.63384796905346, [25.865433811224996, 27.419797516025]) Difference [0.7527764194499897, 3.564680205124982]
PRV 45cm 0 gilbert
Mean (27.398069970184114, [26.67356244565, 28.18309953975]) Difference [-0.00018529120000287946, 2.776812809774991] PRV 45cm 0 2nd skull
Mean (26.622663783041755, [26.094632955374994, 27.218836229399997]) Difference [0.8593674929999944, 3.458514219799995]
PRV 45cm 0 npro
Mean (22.74262953357766, [22.200576709475, 23.297622007025005]) Difference [4.783479303324978, 7.322777977674981]
PRV 45cm 0 gbreaker
Mean (23.531242420735822, [23.125650950649998, 23.991215110175]) Difference [4.021449735649987, 6.4991583156499875]
PRV 45cm 45 no headgear
Mean (25.6024746348144, [24.172775366600003, 27.029188887520004]) PRV 45cm 45 ccc
Mean (25.39719447535409, [23.941374703000005, 26.822381418379997]) Difference [-1.8345421556399901, 2.248237920380004]
PRV 45cm 45 gilbert
Mean (25.72673358058426, [24.22843466606, 27.1856219591]) Difference [-2.16320281246, 1.9405814114799904]
PRV 45cm 45 2nd skull
Mean (25.04259039720532, [23.296280803400002, 26.73740094892]) Difference [-1.6562821561199925, 2.7987250683999996] PRV 45cm 45 npro
Mean (21.598489758191263, [20.342207536319993, 22.842525100079992]) Difference [2.0891153556200104, 5.884383437800009]
PRV 45cm 45 gbreaker
Mean (21.4159062756224, [20.53252909384, 22.309977228039997]) Difference [2.4815608764200006, 5.864311919500002]

PRV 60cm 0 no headgear
Mean (31.556845602615407, [31.01396402329999, 32.123946586624996]) PRV 60cm 0 ccc
Mean (30.31793196709131, [29.820077449400017, 30.82099247715001]) Difference [0.4997272822499752, 2.0262082296249897]
PRV 60cm 0 gilbert
Mean (30.669824123922638, [30.15900418079999, 31.239738477449986]) Difference [0.09869598467500285, 1.6635314144750137]

PRV 60cm 0 2nd skull
Mean (30.36034909330323, [29.85117372667499, 30.91430960602499]) Difference [0.42592467432502157, 1.9742272689000016]
PRV 60cm 0 npro
Mean (26.0678710619861, [25.645323584574992, 26.539669591324994]) Difference [4.770894356150007, 6.206009781500008]
PRV 60cm 0 gbreaker
Mean (26.037373735804135, [25.080888095175006, 26.995837404825007]) Difference [4.400650709224996, 6.639135125574988]
PRV 60cm 45 no headgear
Mean (29.098228124724073, [27.354997688960008, 30.846280849360003]) PRV 60cm 45 ccc
Mean (28.617132162049046, [26.88292538866, 30.298813446019995]) Difference [-1.9699108179199887, 2.919838371440015]
PRV 60cm 45 gilbert
Mean (28.305616347692453, [27.033311396280013, 29.61799181206001]) Difference [-1.430015208059992, 2.9650790901399935]
PRV 60cm 45 2nd skull
Mean (28.587568324613702, [27.105102299920006, 30.076373423400007]) Difference [-1.811800324199988, 2.786922562460012]
PRV 60cm 45 npro
Mean (25.950018016464604, [24.466052921620005, 27.35038424566]) Difference [0.8971771206800101, 5.3892462978400095]
PRV 60cm 45 gbreaker
Mean (24.71390818577454, [23.641336715119994, 25.787279027039993]) Difference [2.2875283995600215, 6.414081553680015]

peak strain cerebrum 15cm 0 no headgear
Mean (0.21460034603460348, [0.208258725, 0.22081362499999999]) peak strain cerebrum 15cm 0 ccc
Mean (0.1867168300830083, [0.18357292499999994, 0.19001502499999995]) Difference [0.020913200000000083, 0.034893000000000035] peak strain cerebrum 15cm 0 gilbert
Mean (0.1906885684568457, [0.18575645000000005, 0.19577650000000002]) Difference [0.015942399999999992, 0.031969524999999915] peak strain cerebrum 15cm 0 2nd skull
Mean (0.18084082513251326, [0.174776, 0.18717539999999996]) Difference [0.024991525000000014, 0.04262999999999999] peak strain cerebrum 15cm 0 npro
Mean (0.15310337523752374, [0.1486544499999999, 0.15773954999999992]) Difference [0.05383485000000007, 0.06930095000000004]
peak strain cerebrum 15cm 0 gbreaker

Mean (0.14856534618461847, [0.14068692500000007,
0.15591207500000007]) Difference [0.056374599999999955,
0.07637219999999992] peak strain cerebrum 15cm 45 no headgear
Mean (0.23465037719771975, [0.22224833999999996,
0.24745853999999998]) peak strain cerebrum 15cm 45 ccc
Mean (0.24782015225522552, [0.2379431999999999,
0.25799423999999993]) Difference [-0.029453659999999989,
0.003098060000000114] peak strain cerebrum 15cm 45 gilbert
Mean (0.24978139421942194, [0.23743540000000007,
0.26207094000000003]) Difference [-0.03274604000000002,
0.0028257000000000004] peak strain cerebrum 15cm 45 2nd skull
Mean (0.24626352263226325, [0.22720908, 0.26492434])
Difference [-0.034101940000000004,
0.011370160000000018] peak strain cerebrum 15cm 45 npro
Mean (0.20496953231323134, [0.19321415999999997,
0.21607922]) Difference [0.012827460000000103,
0.046746659999999995] peak strain cerebrum 15cm 45 gbreaker
Mean (0.20755806892689269, [0.19287042000000004,
0.22176828000000007]) Difference [0.008179699999999991,
0.04645757999999997]

peak strain cerebrum 30cm 0 no headgear
Mean (0.30906769796979694, [0.29479285,
0.32303857499999994]) peak strain cerebrum 30cm 0 ccc
Mean (0.2811505272027203, [0.2763609, 0.28633945])
Difference [0.012547424999999938, 0.042821275]
peak strain cerebrum 30cm 0 gilbert
Mean (0.2916499367936794, [0.2837003249999999,
0.2987627]) Difference [0.0013917499999999486,
0.03378774999999991] peak strain cerebrum 30cm 0 2nd skull
Mean (0.2885595823582358, [0.28056432500000006,
0.29632857500000004]) Difference [0.004392, 0.03689312499999995]
peak strain cerebrum 30cm 0 npro
Mean (0.22990871902190216, [0.22215175000000004,
0.23721315000000007]) Difference [0.06337640000000003,
0.09522887499999999]
peak strain cerebrum 30cm 0 gbreaker
Mean (0.2185969919491949, [0.2091532,
0.22779862500000003]) Difference [0.07352794999999995,
0.10793024999999999]
peak strain cerebrum 30cm 45 no headgear
Mean (0.3440115587158716, [0.3314060800000001,
0.35665622]) peak strain cerebrum 30cm 45 ccc
Mean (0.347671294729473, [0.32399764000000003,
0.37202978000000003]) Difference [-0.03093151999999997,
0.023082040000000036]
peak strain cerebrum 30cm 45 gilbert
Mean (0.3503350378237824, [0.3259509999999998,
0.3761738399999998]) Difference [-0.03460109999999981,
0.02136098000000028] peak strain cerebrum 30cm 45 2nd skull
Mean (0.35685529980998104, [0.33760254, 0.37510886])
Difference [-0.035078499999999777,
0.009945919999999983] peak strain cerebrum 30cm 45 npro
Mean (0.2930006737473747, [0.26995222, 0.31476306])

Difference [0.026038300000000125,
0.07736570000000009] peak strain cerebrum 30cm 45
gbreaker
Mean (0.30606482452245226, [0.2871353399999999,
0.3249216199999998]) Difference [0.01579476000000029,
0.060993080000000345]

peak strain cerebrum 45cm 0 no headgear
Mean (0.3908918204820482, [0.37882210000000005,
0.402935125]) peak strain cerebrum 45cm 0 ccc
Mean (0.35383613556355636, [0.3483145000000002,
0.3596541500000002]) Difference [0.02374594999999992,
0.05032614999999982]
peak strain cerebrum 45cm 0 gilbert
Mean (0.37127023612361243, [0.36171079999999994,
0.3815415499999999]) Difference [0.004237000000000046,
0.03523402499999999] peak strain cerebrum 45cm 0 2nd skull
Mean (0.39016058360836087, [0.3766742999999999,
0.40300214999999995]) Difference [-0.01712962500000007,
0.018925174999999975] peak strain cerebrum 45cm 0 npro
Mean (0.3044509856485648, [0.29455599999999993,
0.3138061499999999]) Difference [0.07096755000000013,
0.1020656250000001]
peak strain cerebrum 45cm 0 gbreaker
Mean (0.30143983648364836, [0.29123037500000004,
0.3111555500000001]) Difference [0.07391247499999994,
0.10518329999999985]
peak strain cerebrum 45cm 45 no headgear
Mean (0.38730855225522554, [0.36666469999999984,
0.4097035399999999]) peak strain cerebrum 45cm 45 ccc
Mean (0.40512762528252827, [0.3861661200000001,
0.42385058]) Difference [-0.046545740000000176,
0.011425739999999893] peak strain cerebrum 45cm 45 gilbert
Mean (0.4111692855685568, [0.39662750000000024,
0.42583604000000014]) Difference [-0.049603020000000296,
0.0030256399999997186] peak strain cerebrum 45cm 45 2nd skull
Mean (0.4019742565056506, [0.3729341, 0.43249808])
Difference [-0.05254746000000003,
0.02243579999999993] peak strain cerebrum 45cm 45
npro
Mean (0.3785086326232623, [0.35553702,
0.4017616599999999]) Difference [-0.02331692000000008,
0.0405001399999999]
peak strain cerebrum 45cm 45 gbreaker
Mean (0.3601840880888089, [0.3387707399999999,
0.3819939599999999]) Difference [-0.003581960000000576,
0.05865420000000004]

peak strain cerebrum 60cm 0 no headgear
Mean (0.40687213106310627, [0.39340254999999996,
0.42195527499999996]) peak strain cerebrum 60cm 0 ccc
Mean (0.376318393039304, [0.36596992500000003,
0.38745660000000004]) Difference [0.012741424999999869,

0.04866222499999995] peak strain cerebrum 60cm 0 gilbert
Mean (0.3883445252525252, [0.37255835000000004,
0.4036913749999999]) Difference [-0.002480725000000024,
0.04016047499999996] peak strain cerebrum 60cm 0 2nd skull
Mean (0.4313871849184919, [0.4143612500000001,
0.44714862500000013]) Difference [-0.04542270000000015,
-0.001797425000000026] peak strain cerebrum 60cm 0 npro
Mean (0.3510221741674167, [0.33926242500000003,
0.36196805000000004]) Difference [0.0382324, 0.07507177500000006]
peak strain cerebrum 60cm 0 gbreaker
Mean (0.3241696773177318, [0.31236342500000003,
0.33566320000000005]) Difference [0.0647009, 0.10165720000000003]
peak strain cerebrum 60cm 45 no headgear
Mean (0.4379289330933094, [0.40742976000000003,
0.4679344999999999]) peak strain cerebrum 60cm 45 ccc
Mean (0.439058101010101, [0.4137616399999999,
0.46510019999999985]) Difference [-0.04171847999999985,
0.038454440000000104] peak strain cerebrum 60cm 45 gilbert
Mean (0.4601527558355835, [0.4243081000000001,
0.49784022000000006]) Difference [-0.07154550000000001,
0.025367259999999836] peak strain cerebrum 60cm 45 2nd skull
Mean (0.4321691795179518, [0.39567522, 0.4697078])
Difference [-0.043310079999999966,
0.05367844000000007] peak strain cerebrum 60cm 45 npro
Mean (0.4072124397239724, [0.3713606599999999,
0.4437524799999999]) Difference [-0.017468759999999986,
0.07762262000000004] peak strain cerebrum 60cm 45 gbreaker
Mean (0.4030415407540754, [0.37357878,
0.43238045999999997]) Difference [-0.008139399999999963,
0.0774408400000001]

peak strain corpus callosum 15cm 0 no headgear
Mean (0.15936596989698967, [0.14392565000000007,
0.17506072500000008]) peak strain corpus callosum 15cm 0 ccc
Mean (0.13997592706270626, [0.12845526750000008,
0.15197197500000006]) Difference [-2.3090000000010324e-05,
0.038917900000000026] peak strain corpus callosum 15cm 0 gilbert
Mean (0.14014694679967998, [0.12635155749999996,
0.154250075]) Difference [-0.0019051674999999019,
0.040068460000000104] peak strain corpus callosum 15cm 0 2nd
skull
Mean (0.11893719428942894, [0.11016535249999998,
0.127580545]) Difference [0.02265654000000008,
0.05855070750000009]
peak strain corpus callosum 15cm 0 npro
Mean (0.11733544329932993, [0.10273694999999997,
0.13154790749999998]) Difference [0.020927850000000095,
0.06364659500000008]
peak strain corpus callosum 15cm 0 gbreaker
Mean (0.10985261486148613, [0.09676678250000002,
0.12261394750000003]) Difference [0.029349550000000058,
0.07006495250000003] peak strain corpus callosum 15cm 45 no headgear

Mean (0.13445614549454943, [0.12624084,
0.14259297999999998]) peak strain corpus callosum 15cm 45 ccc
Mean (0.13128398401840183, [0.12680522,
0.13633401799999997]) Difference [-0.006402899999999986,
0.012483379999999985] peak strain corpus callosum 15cm 45
gilbert
Mean (0.127133301810181, [0.12435592000000001,
0.12978156000000002]) Difference [-0.001418299999999973,
0.015857979999999956] peak strain corpus callosum 15cm 45 2nd skull
Mean (0.12469001656165615, [0.11835029999999999,
0.13072662000000002]) Difference [-0.00046056000000003344,
0.02002685999999997] peak strain corpus callosum 15cm 45 npro
Mean (0.09997150416641663, [0.094921348,
0.10490066599999998]) Difference [0.02480751399999999,
0.04394659200000002]
peak strain corpus callosum 15cm 45 gbreaker
Mean (0.10464862469046904, [0.09841842599999998,
0.11082623399999998]) Difference [0.01961627400000003,
0.04003482200000001]

peak strain corpus callosum 30cm 0 no headgear
Mean (0.23390817816781678, [0.2082327,
0.25960902500000005]) peak strain corpus callosum 30cm 0 ccc
Mean (0.21961001310131015, [0.20224500000000004,
0.238384025]) Difference [-0.01734525000000002,
0.04565075000000006]
peak strain corpus callosum 30cm 0 gilbert
Mean (0.22251109455945595, [0.20286540000000003,
0.243605025]) Difference [-0.02157247499999998,
0.04438029999999994]
peak strain corpus callosum 30cm 0 2nd skull
Mean (0.23412146534653466, [0.21060725000000002,
0.258220275]) Difference [-0.035122049999999974,
0.03499834999999998] peak strain corpus callosum 30cm 0 npro
Mean (0.19050542274227422, [0.16889957500000008,
0.21167242500000003]) Difference [0.010353950000000042,
0.07731942499999994] peak strain corpus callosum 30cm 0 gbreaker
Mean (0.17542017382738273, [0.15382320000000008,
0.19775124500000002]) Difference [0.024488590000000032,
0.09279381999999999] peak strain corpus callosum 30cm 45 no headgear
Mean (0.20019414765476545, [0.18587118000000002,
0.21494745999999998]) peak strain corpus callosum 30cm 45 ccc
Mean (0.18636485328532854, [0.17620851999999998,
0.19727774]) Difference [-0.004343599999999927,
0.031551760000000026] peak strain corpus callosum 30cm 45
gilbert
Mean (0.19635960436043606, [0.18437035999999996,
0.20946987999999997]) Difference [-0.01563263999999989,
0.022786040000000073] peak strain corpus callosum 30cm 45 2nd skull
Mean (0.19457922816281628, [0.18241098000000003,
0.20782617999999997]) Difference [-0.014069599999999936,
0.024710440000000035] peak strain corpus callosum 30cm 45 npro
Mean (0.15287527700770076, [0.14575799999999994,
0.16014137999999994]) Difference [0.03137972000000003,

0.06356812000000003]
peak strain corpus callosum 30cm 45 gbreaker
Mean (0.1681466709070907, [0.16206822000000004,
0.17397592000000003]) Difference [0.016593979999999973,
0.04771491999999994]

peak strain corpus callosum 45cm 0 no headgear
Mean (0.32881546984698473, [0.2852128500000002,
0.3729243000000001]) peak strain corpus callosum 45cm 0 ccc
Mean (0.297703203120312, [0.261944575, 0.3338762])
Difference [-0.025756949999999973,
0.08834542500000006] peak strain corpus callosum 45cm 0
gilbert
Mean (0.27429107415741577, [0.23176882500000018,
0.31538685000000016]) Difference [-0.005942524999999976,
0.1161739249999999]
peak strain corpus callosum 45cm 0 2nd skull
Mean (0.32586063141314137, [0.27521294999999996,
0.3741882749999999]) Difference [-0.06281869999999992,
0.07100747500000001]
peak strain corpus callosum 45cm 0 npro
Mean (0.27003584148414844, [0.231381275, 0.30765615])
Difference [0.001138400000000192,
0.11794232500000011] peak strain corpus callosum 45cm 0
gbreaker
Mean (0.25086068106810683, [0.21944189999999986,
0.2827305749999999]) Difference [0.024055525000000327,
0.13256127500000023] peak strain corpus callosum 45cm 45 no headgear
Mean (0.2257553273327333, [0.21357870000000004,
0.23922793999999997]) peak strain corpus callosum 45cm 45 ccc
Mean (0.2465053633763376, [0.22640116000000005,
0.2665619]) Difference [-0.04449367999999996,
0.003941339999999995] peak strain corpus callosum 45cm 45
gilbert
Mean (0.25576735057505745, [0.24038976000000006,
0.27206094000000003]) Difference [-0.050553260000000017,
-0.009525460000000131] peak strain corpus callosum 45cm 45 2nd skull
Mean (0.24072115503550356, [0.22419478000000004,
0.25891688]) Difference [-0.0374538, 0.006372139999999971]
peak strain corpus callosum 45cm 45 npro
Mean (0.2027778715871587, [0.19304443999999996,
0.21311781999999999]) Difference [0.00666143999999997,
0.039709239999999944] peak strain corpus callosum 45cm 45 gbreaker
Mean (0.19883383114311434, [0.1895703800000001,
0.20904894000000007]) Difference [0.010608599999999975,
0.04355065999999991]

peak strain corpus callosum 60cm 0 no headgear
Mean (0.27022135523552354, [0.25176307500000006,
0.2889963]) peak strain corpus callosum 60cm 0 ccc
Mean (0.2993288637863786, [0.27737687499999997,
0.32226174999999996]) Difference [-0.05800374999999994,
7.527500000006904e-05] peak strain corpus callosum 60cm 0 gilbert

Mean (0.27556382728272827, [0.256502625,
0.29501742499999994]) Difference [-0.03237647499999999,
0.021690775000000023]
peak strain corpus callosum 60cm 0 2nd skull
Mean (0.3653726116111611, [0.31236862500000007,
0.41773227499999993]) Difference [-0.15005427500000001,
-0.03745260000000003] peak strain corpus callosum 60cm 0 npro
Mean (0.31132785688568854, [0.2703127250000001,
0.35192025]) Difference [-0.08559632499999993,
0.0048404499999998435] peak strain corpus callosum 60cm 0
gbreaker
Mean (0.22751940014001398, [0.2170753250000001,
0.2377660750000001]) Difference [0.021439774999999925,
0.06490564999999984] peak strain corpus callosum 60cm 45 no headgear
Mean (0.3022025170517052, [0.26611038000000004,
0.33624602000000003]) peak strain corpus callosum 60cm 45 ccc
Mean (0.27648523640364037, [0.25521780000000005,
0.2986198000000001]) Difference [-0.015934059999999903, 0.06567686]
peak strain corpus callosum 60cm 45 gilbert
Mean (0.26797191979197915, [0.24689761999999998,
0.2904717399999999]) Difference [-0.008007019999999865,
0.07428228000000012] peak strain corpus callosum 60cm 45 2nd skull
Mean (0.2715199473947395, [0.24603927999999997,
0.2984302999999999]) Difference [-0.013747979999999799,
0.07327688000000014] peak strain corpus callosum 60cm 45 npro
Mean (0.2400577388538854, [0.22532867999999995,
0.25604121999999996]) Difference [0.023330760000000242,
0.09906182000000013] peak strain corpus callosum 60cm 45 gbreaker
Mean (0.23943091029102911, [0.22464996000000004,
0.25524700000000006]) Difference [0.023771780000000006,
0.09998434000000009]

peak strain brainstem 15cm 0 no headgear
Mean (0.26553705710571057, [0.24512000000000012,
0.2849269750000001]) peak strain brainstem 15cm 0 ccc
Mean (0.22505268721872188, [0.2090738000000001,
0.24080277500000008]) Difference [0.014120425000000013,
0.06566510000000006]
peak strain brainstem 15cm 0 gilbert
Mean (0.22947485573557355, [0.21036522500000007,
0.24806587500000013]) Difference [0.008312824999999954,
0.06335739999999992] peak strain brainstem 15cm 0 2nd skull
Mean (0.20551608590859086, [0.19867694999999996,
0.21180077499999994]) Difference [0.03821812500000009,
0.08067810000000022]
peak strain brainstem 15cm 0 npro
Mean (0.19007922812281225, [0.172356825,
0.20686674999999996]) Difference [0.0485372250000002,
0.10207672500000017]
peak strain brainstem 15cm 0 gbreaker
Mean (0.17116202830283028, [0.15717442500000006,
0.1846743]) Difference [0.06932910000000007,

0.11846635000000008]
peak strain brainstem 15cm 45 no headgear
Mean (0.25702869646964693, [0.23748068,
0.27702496000000004]) peak strain brainstem 15cm 45 ccc
Mean (0.18688722400240024, [0.18106725999999998, 0.19279243999999998])
Difference [0.049685840000000106, 0.0910269200000001]
peak strain brainstem 15cm 45 gilbert
Mean (0.18287577605760574, [0.17638957999999996,
0.1888246799999999]) Difference [0.05355754000000003,
0.09491056000000009]
peak strain brainstem 15cm 45 2nd skull
Mean (0.23582363840384035, [0.22212364000000007,
0.24856584000000007]) Difference [-0.0024702400000000055,
0.04550625999999994] peak strain brainstem 15cm 45 npro
Mean (0.18623174501450143, [0.17949436,
0.19244183999999995]) Difference [0.049978960000000086,
0.09236362000000005] peak strain brainstem 15cm 45 gbreaker
Mean (0.19659465646564658, [0.18235594000000005,
0.20994894000000006]) Difference [0.03622272000000003,
0.08567036000000003]

peak strain brainstem 30cm 0 no headgear
Mean (0.37257731318131815, [0.34879194999999996,
0.39474042499999995]) peak strain brainstem 30cm 0 ccc
Mean (0.3373366593659366, [0.317575325, 0.356618275])
Difference [0.003962275000000015, 0.0654833249999999]
peak strain brainstem 30cm 0 gilbert
Mean (0.3486043237323732, [0.3290891, 0.36842545])
Difference [-0.006961950000000057,
0.05435389999999993] peak strain brainstem 30cm 0 2nd
skull
Mean (0.3498894635963596, [0.32908859999999995,
0.3698312499999999]) Difference [-0.008952224999999918,
0.05340220000000008] peak strain brainstem 30cm 0 npro
Mean (0.2734495805580558, [0.25026625000000013,
0.29489355000000006]) Difference [0.06651819999999989, 0.132133025]
peak strain brainstem 30cm 0 gbreaker
Mean (0.2512677541754176, [0.23364239999999997,
0.2681715]) Difference [0.0924333749999999, 0.15018335]
peak strain brainstem 30cm 45 no headgear
Mean (0.3565499825982598, [0.32888787999999997,
0.38376103999999994]) peak strain brainstem 30cm 45 ccc
Mean (0.25724059977997804, [0.24390278000000004,
0.27076804]) Difference [0.06874009999999994,
0.12929659999999993]
peak strain brainstem 30cm 45 gilbert
Mean (0.26996995191519146, [0.25654646,
0.28392738000000006]) Difference [0.05544579999999996,
0.11675265999999992]
peak strain brainstem 30cm 45 2nd skull
Mean (0.3428449272527253, [0.3203281399999999,
0.3651920599999998]) Difference [-0.02172851999999996,
0.049609280000000124] peak strain brainstem 30cm 45 npro
Mean (0.28214258821882193, [0.2688038000000001,

0.29478694000000005]) Difference [0.043757279999999885,
0.10464487999999988] peak strain brainstem 30cm 45 gbreaker
Mean (0.2958944226422642, [0.27529368000000004,
0.31573426000000004]) Difference [0.026400539999999903,
0.0957333199999999]
peak strain brainstem 45cm 0 no headgear
Mean (0.4851650134013401, [0.4399122500000001,
0.5300247250000001]) peak strain brainstem 45cm 0 ccc
Mean (0.4335417521752175, [0.40202079999999996,
0.4647213]) Difference [-0.0033175999999997957,
0.10645570000000013] peak strain brainstem 45cm 0 gilbert
Mean (0.4409874288428844, [0.40322135000000003,
0.4775880000000001]) Difference [-0.014044074999999809,
0.10342632500000001] peak strain brainstem 45cm 0 2nd skull
Mean (0.4828920800580058, [0.432165275,
0.5310655750000001]) Difference [-0.0649007499999998,
0.07045225000000016]
peak strain brainstem 45cm 0 npro
Mean (0.378854895489549, [0.33617592500000004,
0.4196786]) Difference [0.0453670500000001,
0.1682608000000001]
peak strain brainstem 45cm 0 gbreaker
Mean (0.36123303480348035, [0.3387464250000001,
0.38371545000000007]) Difference [0.07342722500000012,
0.17395339999999992]
peak strain brainstem 45cm 45 no headgear
Mean (0.3333776932893289, [0.3139575, 0.35296374])
peak strain brainstem 45cm 45 ccc
Mean (0.4034407839583958, [0.3670140600000001,
0.43851941999999994]) Difference [-0.1101835599999999,
-0.02810221999999986]
peak strain brainstem 45cm 45 gilbert
Mean (0.41651320744074405, [0.38442034000000014,
0.44932228000000013]) Difference [-0.12075490000000014,
-0.04479200000000019] peak strain brainstem 45cm 45 2nd skull
Mean (0.33822972485248526, [0.31004356,
0.36653716000000003]) Difference [-0.03925167999999994,
0.029164600000000027] peak strain brainstem 45cm 45 npro
Mean (0.2759610051405141, [0.26442000000000004,
0.28813366]) Difference [0.03448626000000013,
0.08033623999999998]
peak strain brainstem 45cm 45 gbreaker
Mean (0.2782265579757976, [0.26407534000000005,
0.29160614]) Difference [0.03167947999999998,
0.07951690000000003]

peak strain brainstem 60cm 0 no headgear
Mean (0.4479322431243124, [0.4315699000000001,
0.4621077000000002]) peak strain brainstem 60cm 0 ccc
Mean (0.44109859715971594, [0.41733647499999993,
0.462014725]) Difference [-0.020216849999999828,
0.03455935000000017] peak strain brainstem 60cm 0 gilbert
Mean (0.4435775341534153, [0.4213299999999999,
0.46379327499999995]) Difference [-0.021886449999999846,

0.030861500000000215] peak strain brainstem 60cm 0 2nd skull
Mean (0.5312278687368737, [0.476963625,
0.5830938749999999]) Difference [-0.13733677499999988,
-0.027212225000000034] peak strain brainstem 60cm 0 npro
Mean (0.4323415698069807, [0.3865767750000015,
0.4766787500000001]) Difference [-0.0311484999999999, 0.063900175]
peak strain brainstem 60cm 0 gbreaker
Mean (0.36079914111411143, [0.34731115,
0.3746140499999999]) Difference [0.06573347500000017,
0.1070503500000002]
peak strain brainstem 60cm 45 no headgear
Mean (0.43054100242024207, [0.37147280000000005,
0.48402159999999994]) peak strain brainstem 60cm 45 ccc
Mean (0.38771631059105915, [0.35277938,
0.42272909999999986]) Difference [-0.025615159999999818,
0.10678488000000008] peak strain brainstem 60cm 45 gilbert
Mean (0.3753705725372537, [0.34863620000000006,
0.40277500000000005]) Difference [-0.009217119999999981,
0.1146555199999998]
peak strain brainstem 60cm 45 2nd skull
Mean (0.35972022734273434, [0.33892387999999996,
0.38151995999999994]) Difference [0.00835822000000011,
0.12814970000000003]
peak strain brainstem 60cm 45 npro
Mean (0.3416391541954195, [0.31508713999999993,
0.3676860399999999]) Difference [0.023918160000000188,
0.14857521999999995] peak strain brainstem 60cm 45 gbreaker
Mean (0.31261520632063206, [0.30012428,
0.32506747999999996]) Difference [0.058296719999999996,
0.17313294]

CSDM 15 corpus callosum 15cm 0 no headgear
Mean (2.2735435536053603, [1.099866950000001,
3.3304220749999995]) CSDM 15 corpus callosum 15cm 0 ccc
Mean (0.7615962595259527, [0.5026250000000001,
1.0023270000000004]) Difference [0.3306584500000005,
2.6165589999999996]
CSDM 15 corpus callosum 15cm 0 gilbert
Mean (1.1411162639763979, [0.5346082500000009,
1.6667486749999996]) Difference [-0.1531186749999982, 2.3761609]
CSDM 15 corpus callosum 15cm 0 2nd skull
Mean (0.29138000000000003, [0.29138000000000003,
0.29138000000000003]) Difference [0.8084869500000011,
3.0390420749999993]
CSDM 15 corpus callosum 15cm 0 npro
Mean (0.6035517047704771, [0.3583959999999997,
0.8479129999999998]) Difference [0.4895104749999999,
2.7681417499999994]
CSDM 15 corpus callosum 15cm 0 gbreaker
Mean (0.40730513736373636, [0.28846499999999986,
0.5069984999999999]) Difference [0.6917870250000027,
2.9300041999999986]

CSDM 15 corpus callosum 15cm 45 no headgear
Mean (0.5931668501250125, [0.31818836000000006, 0.8216744]) CSDM 15 corpus callosum 15cm 45 ccc
Mean (0.29138000000000003, [0.29138, 0.29138])
Difference [0.026808360000000087, 0.5302944]
CSDM 15 corpus callosum 15cm 45 gilbert
Mean (0.29138000000000003, [0.29138, 0.29138])
Difference [0.026808360000000087, 0.5302944]
CSDM 15 corpus callosum 15cm 45 2nd skull
Mean (0.30898095685568555, [0.282056, 0.32634319999999994]) Difference [0.009261220000000958, 0.51631084]
CSDM 15 corpus callosum 15cm 45 npro
Mean (0.29138000000000003, [0.29138, 0.29138])
Difference [0.026808360000000087, 0.5302944]
CSDM 15 corpus callosum 15cm 45 gbreaker
Mean (0.29138000000000003, [0.29138, 0.29138])
Difference [0.026808360000000087, 0.5302944]

CSDM 15 corpus callosum 30cm 0 no headgear
Mean (9.231546190469047, [6.872011450000002, 11.790727000000002]) CSDM 15 corpus callosum 30cm 0 ccc
Mean (7.329264371737174, [5.648309875000001, 9.256974175000002]) Difference [-1.1178510749999937, 4.969550625000002]
CSDM 15 corpus callosum 30cm 0 gilbert
Mean (7.968628842884288, [6.136350174999999, 10.042314975]) Difference [-1.8722096249999947, 4.452493500000003]
CSDM 15 corpus callosum 30cm 0 2nd skull
Mean (8.473841132713272, [6.410179425000001, 10.763400049999998]) Difference [-2.499995549999997, 4.0677458750000035]
CSDM 15 corpus callosum 30cm 0 npro
Mean (4.3609794431443145, [2.693617050000004, 5.933853300000001]) Difference [2.022061425000002, 7.910983325000002]
CSDM 15 corpus callosum 30cm 0 gbreaker
Mean (3.4091630291029102, [1.9230919750000002, 4.790355474999999]) Difference [3.028755925000004, 8.768991250000003]
CSDM 15 corpus callosum 30cm 45 no headgear
Mean (6.574943429982999, [4.321687739999998, 8.844058539999999]) CSDM 15 corpus callosum 30cm 45 ccc
Mean (5.418509904430444, [3.737528240000003, 7.035147479999999]) Difference [-1.5991383800000007, 3.962930099999997]
CSDM 15 corpus callosum 30cm 45 gilbert
Mean (7.422041830023001, [5.437020560000002, 9.46976212]) Difference [-3.9174405000000014, 2.110948619999997]
CSDM 15 corpus callosum 30cm 45 2nd skull
Mean (5.972133437983798, [3.6875897199999983, 8.129372159999997]) Difference [-2.5222099, 3.814801599999999]

CSDM 15 corpus callosum 30cm 45 npro
Mean (0.7515560581258126, [0.4708691800000002, 0.99073378]) Difference [3.541896699999999, 8.115381379999999]
CSDM 15 corpus callosum 30cm 45 gbreaker
Mean (2.160493116551655, [1.4277439000000007, 2.83454434]) Difference [2.0034386399999997, 6.755475899999997]

CSDM 15 corpus callosum 45cm 0 no headgear
Mean (16.255929745974598, [13.359408749999997, 19.546982499999988]) CSDM 15 corpus callosum 45cm 0 ccc
Mean (13.578302132213222, [10.828799750000002, 16.771540500000004]) Difference [-1.6101212500000068, 6.947995249999982]
CSDM 15 corpus callosum 45cm 0 gilbert
Mean (12.592623976897691, [9.69681925, 15.722740499999997]) Difference [-0.5872657500000109, 8.01601724999999]
CSDM 15 corpus callosum 45cm 0 2nd skull
Mean (15.394538011801181, [12.85549425, 18.208127499999996]) Difference [-3.22115050000001, 4.985648249999991]
CSDM 15 corpus callosum 45cm 0 npro
Mean (10.310811641164115, [7.670460000000002, 13.17890525]) Difference [1.8366172499999944, 10.137078124999992]
CSDM 15 corpus callosum 45cm 0 gbreaker
Mean (9.430959761276126, [7.0191589000000025, 11.991778475]) Difference [2.8421379749999964, 10.84818292499999]
CSDM 15 corpus callosum 45cm 45 no headgear
Mean (12.548228512571256, [9.434628640000005, 15.7482613]) CSDM 15 corpus callosum 45cm 45 ccc
Mean (11.701380338433843, [8.4367682, 15.031696999999994]) Difference [-3.7658435599999924, 5.391917400000003]
CSDM 15 corpus callosum 45cm 45 gilbert
Mean (12.796503953595359, [9.914916000000003, 15.7693408]) Difference [-4.594571479999999, 3.9817085999999975]
CSDM 15 corpus callosum 45cm 45 2nd skull
Mean (12.133908500450046, [8.6490751, 15.761133859999997]) Difference [-4.371967659999996, 5.113356020000002]
CSDM 15 corpus callosum 45cm 45 npro
Mean (7.037624849044905, [5.1654998199999955, 8.921898419999996]) Difference [1.82716932000001, 9.149313180000004]
CSDM 15 corpus callosum 45cm 45 gbreaker
Mean (7.247824356075606, [5.547729820000001, 8.959208839999999]) Difference [1.772616580000001, 8.880011199999998]

CSDM 15 corpus callosum 60cm 0 no headgear
Mean (17.314234989498953, [15.27075700000001, 19.57620525]) CSDM 15 corpus callosum 60cm 0 ccc
Mean (17.013736114111413, [14.910915999999997, 19.40727799999999]) Difference [-2.8628374999999964, 3.440170750000003]
CSDM 15 corpus callosum 60cm 0 gilbert
Mean (16.705734337433743, [14.698424500000002, 19.015305249999997]) Difference [-2.475461999999991, 3.6775237499999993]
CSDM 15 corpus callosum 60cm 0 2nd skull
Mean (18.103007458245823, [15.626313500000002, 21.0169675]) Difference [-4.315327499999989, 2.686999249999997]
CSDM 15 corpus callosum 60cm 0 npro
Mean (13.352636845184518, [10.7124, 16.311408]) Difference [0.38300999999999785, 7.460971250000002]
CSDM 15 corpus callosum 60cm 0 gbreaker
Mean (9.27276608860886, [7.700972000000004, 10.96888775]) Difference [5.3641617500000045, 10.784119500000001]
CSDM 15 corpus callosum 60cm 45 no headgear
Mean (15.50038599019902, [12.149707400000015, 19.0759226]) CSDM 15 corpus callosum 60cm 45 ccc
Mean (15.10839302570257, [11.652563800000003, 18.804266000000002]) Difference [-4.628228, 5.360198]
CSDM 15 corpus callosum 60cm 45 gilbert
Mean (14.965225733773377, [11.797087400000002, 18.328772999999998]) Difference [-4.2436385999999935, 5.271727200000004]
CSDM 15 corpus callosum 60cm 45 2nd skull
Mean (15.035118926292629, [11.424247599999994, 18.832156799999996]) Difference [-4.624758199999996, 5.532924200000002]
CSDM 15 corpus callosum 60cm 45 npro
Mean (11.713081719971996, [8.814459800000003, 14.741327920000002]) Difference [-0.7820508199999985, 8.311423600000001]
CSDM 15 corpus callosum 60cm 45 gbreaker
Mean (11.91589819269927, [9.046565920000004, 14.954558820000003]) Difference [-0.9965132199999989, 8.167995920000001]

CSDM 15 brainstem 15cm 0 no headgear
Mean (1.3283350579557955, [1.0776634250000003, 1.5629896249999995]) CSDM 15 brainstem 15cm 0 ccc
Mean (0.463332401740174, [0.37359299999999995, 0.552918025]) Difference [0.59863095, 1.1216611249999997]
CSDM 15 brainstem 15cm 0 gilbert
Mean (0.5194863166816682, [0.39029599999999975,

0.6435097749999996]) Difference [0.5274200249999998,
1.0838620750000003]
CSDM 15 brainstem 15cm 0 2nd skull
Mean (0.40346558545854583, [0.3551345, 0.453586025])
Difference [0.6698383749999999, 1.1656170249999995]
CSDM 15 brainstem 15cm 0 npro
Mean (0.19592471156115612, [0.10812234500000006,
0.27865760250000005]) Difference [0.8675784250000005,
1.3880141349999997]
CSDM 15 brainstem 15cm 0 gbreaker
Mean (0.14566830214021403, [0.05889599500000006,
0.22508139249999984]) Difference [0.9194278975000003,
1.4372417124999997]
CSDM 15 brainstem 15cm 45 no headgear
Mean (1.0022941832943293, [0.7284762780000011,
1.269339962]) CSDM 15 brainstem 15cm 45 ccc
Mean (0.2259380119811981, [0.15682186199999998,
0.28692048400000003]) Difference [0.4943392480000006,
1.0520383960000002]
CSDM 15 brainstem 15cm 45 gilbert
Mean (0.1596509429022902, [0.1350218, 0.18354464])
Difference [0.5667708700000003, 1.10833256]
CSDM 15 brainstem 15cm 45 2nd skull
Mean (0.5273286911491148, [0.38889114000000014,
0.6554252200000001]) Difference [0.16876211200000002,
0.7763630920000002]
CSDM 15 brainstem 15cm 45 npro
Mean (0.09486761943394338, [0.06399475799999996,
0.12306572199999996]) Difference [0.6300976439999999, 1.175844524]
CSDM 15 brainstem 15cm 45 gbreaker
Mean (0.2305470816561656, [0.1364282340000001,
0.31645604600000005]) Difference [0.48452496200000006,
1.0548500600000001]
CSDM 15 brainstem 30cm 0 no headgear
Mean (7.220183474847486, [5.810402000000002,
8.67353]) CSDM 15 brainstem 30cm 0 ccc
Mean (4.85476097359736, [4.440940000000001,
5.284815250000002]) Difference [0.8745919999999985,
3.8916372499999987]
CSDM 15 brainstem 30cm 0 gilbert
Mean (5.8712353655365535, [5.268941249999999,
6.4425634999999986]) Difference [-0.17844050000000156,
2.946550750000001] CSDM 15 brainstem 30cm 0 2nd skull
Mean (5.723877382738273, [5.11511625, 6.3713055])
Difference [-0.06859875000000004,
3.0986399999999996] CSDM 15 brainstem 30cm 0 npro
Mean (1.9525535648564856, [1.4565597500000003,
2.3997705000000007]) Difference [3.781627000000002,
6.8225437499999995]
CSDM 15 brainstem 30cm 0 gbreaker
Mean (1.5643744649964995, [1.1831978499999996,
1.932184675]) Difference [4.196554800000003,
7.1712861750000005]
CSDM 15 brainstem 30cm 45 no headgear
Mean (4.81292298429843, [3.7348454,

5.8622423999999995]) CSDM 15 brainstem 30cm 45 ccc
Mean (2.0508265608160814, [1.6391997120000008,
2.471154904]) Difference [1.6075445359999994,
3.893150263999999]
CSDM 15 brainstem 30cm 45 gilbert
Mean (3.176033592719272, [2.4781602,
3.8755137599999996]) Difference [0.3410920799999997,
2.887526279999999]
CSDM 15 brainstem 30cm 45 2nd skull
Mean (3.853749545754576, [3.1110816000000012,
4.541492]) Difference [-0.3509861999999999,
2.240358599999997]
CSDM 15 brainstem 30cm 45 npro
Mean (1.283756886648665, [1.09915258, 1.43739926])
Difference [2.43110774, 4.602023139999999]
CSDM 15 brainstem 30cm 45 gbreaker
Mean (1.9382380078807884, [1.4725782000000005,
2.3713080200000007]) Difference [1.699703839999998,
4.042947999999997]

CSDM 15 brainstem 45cm 0 no headgear
Mean (15.371951241124115, [13.654169750000005,
17.108091]) CSDM 15 brainstem 45cm 0 ccc
Mean (12.03573192569257, [10.993315,
13.040614999999995]) Difference [1.3308120000000097,
5.352543250000008]
CSDM 15 brainstem 45cm 0 gilbert
Mean (12.278942727772778, [10.652200750000004,
13.917010500000002]) Difference [0.736542, 5.501235249999999]
CSDM 15 brainstem 45cm 0 2nd skull
Mean (12.847881593159315, [11.661209749999998,
14.00320325]) Difference [0.4350807500000071,
4.6281565000000064]
CSDM 15 brainstem 45cm 0 npro
Mean (6.8142605230523055, [5.619593500000002,
7.929057750000001]) Difference [6.486435000000004,
10.665540500000004]
CSDM 15 brainstem 45cm 0 gbreaker
Mean (6.193256311631163, [5.7673567499999985,
6.660563749999999]) Difference [7.413801250000002,
10.989805250000007]
CSDM 15 brainstem 45cm 45 no headgear
Mean (8.256234463446344, [6.196677000000003,
10.174533]) CSDM 15 brainstem 45cm 45 ccc
Mean (8.277663174717471, [6.526739600000001,
9.9767964]) Difference [-2.678878599999995,
2.5928428000000006]
CSDM 15 brainstem 45cm 45 gilbert
Mean (9.439620526452645, [7.444436000000001,
11.4015482]) Difference [-4.062659, 1.5825368000000006]
CSDM 15 brainstem 45cm 45 2nd skull
Mean (7.346015481148115, [5.343109499999999,
9.317904799999999]) Difference [-1.9592190999999963,
3.6940789000000027] CSDM 15 brainstem 45cm 45 npro

Mean (3.036282115571557, [2.4184307, 3.64772184])
Difference [3.080711400000003, 7.239132800000002]
CSDM 15 brainstem 45cm 45 gbreaker
Mean (3.147762215821582, [2.4619875200000014, 3.79824544]) Difference [2.961941720000002, 7.147841280000001]

CSDM 15 brainstem 60cm 0 no headgear
Mean (16.382284943494348, [15.44919475, 17.31892525]) CSDM 15 brainstem 60cm 0 ccc
Mean (15.020975301030102, [14.351269750000007, 15.736640250000006]) Difference [0.20209724999999423, 2.538666749999991]
CSDM 15 brainstem 60cm 0 gilbert
Mean (15.249071473147314, [14.573611249999995, 15.995950499999996]) Difference [-0.02986124999999573, 2.3110554999999997] CSDM 15 brainstem 60cm 0 2nd skull
Mean (16.02189401690169, [14.766164750000002, 17.306615]) Difference [-1.197294250000002, 1.9560114999999947]
CSDM 15 brainstem 60cm 0 npro
Mean (10.45566594409441, [9.150688750000004, 11.710649]) Difference [4.360916999999998, 7.544898999999998]
CSDM 15 brainstem 60cm 0 gbreaker
Mean (7.868699532453245, [6.848537250000003, 8.870532750000002]) Difference [7.140280749999999, 9.915663749999997]
CSDM 15 brainstem 60cm 45 no headgear
Mean (12.531406191019101, [10.20783180000001, 14.874890000000002]) CSDM 15 brainstem 60cm 45 ccc
Mean (11.020363754775476, [8.385994000000002, 13.63238]) Difference [-2.0102065999999903, 4.978974000000004]
CSDM 15 brainstem 60cm 45 gilbert
Mean (9.937202273427342, [8.063914200000003, 11.811640600000002]) Difference [-0.4220577999999957, 5.597102400000002]
CSDM 15 brainstem 60cm 45 2nd skull
Mean (9.408014111411141, [7.8396677999999955, 11.088607999999997]) Difference [0.26652320000000707, 5.986214000000002]
CSDM 15 brainstem 60cm 45 npro
Mean (6.296874806280628, [5.2053088, 7.398778999999998]) Difference [3.6974942000000066, 8.827031200000004] CSDM 15 brainstem 60cm 45 gbreaker
Mean (5.009091339533954, [4.1828554, 5.7974722])
Difference [5.0781548000000125, 10.018311400000005]
